# Determining by Raman spectroscopy the average thickness and *N*-layer-specific surface coverages of MoS_2_ thin films with domains much smaller than the laser spot size

**DOI:** 10.3762/bjnano.15.26

**Published:** 2024-03-07

**Authors:** Felipe Wasem Klein, Jean-Roch Huntzinger, Vincent Astié, Damien Voiry, Romain Parret, Houssine Makhlouf, Sandrine Juillaguet, Jean-Manuel Decams, Sylvie Contreras, Périne Landois, Ahmed-Azmi Zahab, Jean-Louis Sauvajol, Matthieu Paillet

**Affiliations:** 1 Laboratoire Charles Coulomb, Université de Montpellier, CNRS, F-34095, Montpellier, France; 2 Annealsys, 139 Rue des Walkyries, 34000 Montpellier, France; 3 Institut Européen des Membranes, IEM, UMR 5635, Université Montpellier, ENSCM, CNRS, Montpellier, France; 4 Aix Marseille Université, CNRS, CINAM, UMR 7325, Campus de Luminy, 13288, Marseille, France

**Keywords:** molybdenum disulfide, number of layers, Raman spectroscopy, thin film, transition metal dichalcogenides

## Abstract

Raman spectroscopy is a widely used technique to characterize nanomaterials because of its convenience, non-destructiveness, and sensitivity to materials change. The primary purpose of this work is to determine via Raman spectroscopy the average thickness of MoS_2_ thin films synthesized by direct liquid injection pulsed-pressure chemical vapor deposition (DLI-PP-CVD). Such samples are constituted of nanoflakes (with a lateral size of typically 50 nm, i.e., well below the laser spot size), with possibly a distribution of thicknesses and twist angles between stacked layers. As an essential preliminary, we first reassess the applicability of different Raman criteria to determine the thicknesses (or layer number, *N*) of MoS_2_ flakes from measurements performed on reference samples, namely well-characterized mechanically exfoliated or standard chemical vapor deposition MoS_2_ large flakes deposited on 90 ± 6 nm SiO_2_ on Si substrates. Then, we discuss the applicability of the same criteria for significantly different DLI-PP-CVD MoS_2_ samples with average thicknesses ranging from sub-monolayer up to three layers. Finally, an original procedure based on the measurement of the intensity of the layer breathing modes is proposed to evaluate the surface coverage for each *N* (i.e., the ratio between the surface covered by exactly *N* layers and the total surface) in DLI-PP-CVD MoS_2_ samples.

## Introduction

The advent of two-dimensional (2D) layered materials beyond graphene has initiated a new field of research [1–3]. In the family of 2D layered structures, transition metal dichalcogenides (TMDs) have attracted considerable attention from academia and regarding potential applications [4–9] because of a number of remarkable properties [10–12]. Particularly, it was found that the properties of layered TMDs drastically change when their thickness is reduced to a monolayer [13–14]. Layered TMD structures have a graphite-like structure with each graphene sheet replaced with an X–M–X or MX_2_ triatomic layer, where X is a chalcogen atom (e.g., sulfur, selenium, or tellurium) and M is a transition metal atom (e.g., molybdenum or tungsten) [10].

Among the layered TMD materials, molybdenum disulfide, MoS_2_, is of particular interest in optoelectronic applications because of its transition to a direct bandgap semiconductor with very high photoluminescence quantum yield when thinned down to a monolayer [13–17]. Its unique electronic and optical properties could provide an edge in many future applications.

The multilayers MoS_2_ structures are of the most common 2Hc type, where atomic layers are arranged in such way that the stacking between two adjacent layers corresponds to a twist angle of θ = 60°, and any Mo atom is sitting on top of two S atoms of the adjacent layers [18–19]. However, during the synthesis process (e.g., chemical vapor deposition (CVD) synthesis) or when using precise transfer or AFM tip manipulation techniques [20], twisted MoS_2_ can be formed with two adjacent layers stacked with a relative twist angle (θ) varying from 0 to 60°. Such twisted-layered MoS_2_ structures can exhibit a variety of interesting physical properties including unconventional super conductivity [21–22], non-linear optics [23–24], and moiré excitons [25].

Because the properties of MoS_2_ flakes are first a function of their thickness, or layer number (*N*), it is of a primary importance to determine the *N* of MoS_2_ flakes, including twisted MoS_2_ flakes and defective MoS_2_ flakes, synthesized by different ways. Independently of the structural organization between adjacent layers, a MoS_2_ flake is usually named *N*L-MoS_2_, or simply *N*L, with *N* being the number of MoS_2_ triatomic layers, which defines the thickness of the flake.

Several optical techniques have been developed to identify the *N* of MoS_2_ flakes produced by different methods. Among these techniques, Raman spectroscopy is widely used thanks to its convenience, non-destructiveness, and sensitivity to materials change, including strain, temperature, doping, and defects [26]. Concerning the characterization of MoS_2_ flakes, different information can be derived from the measurement of the Raman features (frequencies, linewidths, and intensities) of intralayer phonon modes as well as those of the interlayer modes, the so-called layer breathing (LB) modes and shear (S) modes.

Recently, we have developed the reproducible direct growth of wafer-scale MoS_2_ thin films on SiO_2_/Si substrates by direct liquid injection pulsed-pressure chemical vapor deposition (DLI-PP-CVD) using low-toxicity precursors [27]. Such MoS_2_ thin films showed good stoichiometry (Mo/S = 1.94–1.95) and the potential for high photoluminescence quantum yield. However, atomic force microscopy revealed that they are constituted of nanoflakes (with a lateral size of typically 50 nm) with possibly a distribution of thicknesses. Furthermore, depending on the synthesis conditions, the MoS_2_ surface coverage can be incomplete, and the thin film average thickness can vary. These samples thus have characteristics, especially thickness inhomogeneities smaller than the laser spot size, that differ from the ones used to establish Raman spectroscopy-based MoS_2_ layer counting methods [26,28–33]. In this context, the primary purpose of this work is to develop and validate an approach for determining the average thickness of such sub-laser spot size inhomogeneous MoS_2_ thin films using Raman spectroscopy.

First, we reassess here as a ground work the information that can be derived from the Raman spectra of MoS_2_ flakes for the evaluation of their thickness, *N*. Different Raman criteria for the determination of the thicknesses of MoS_2_ flakes are first recollected; after the specification of the experimental protocol, domains and limits of application of these criteria are precisely defined from measurements performed on reference samples. These samples are well-characterized, either mechanically exfoliated or standard CVD MoS_2_ large flakes deposited on 90 ± 6 nm SiO_2_ on Si substrates. Then, we determine which Raman information is relevant to estimate the average thickness of MoS_2_ samples produced by the DLI-PP-CVD method, which are constituted of nanoflakes and, thus, significantly different from the reference samples. Finally, an original procedure based on the layer breathing mode intensities is proposed to evaluate the surface coverage for each *N*, that is, the ratio between the surface covered by exactly *N* layers and the total surface, in DLI-PP-CVD samples.

## Results and Discussion

### Experimental procedure

To define a robust experimental Raman protocol to evaluate the thickness of a MoS_2_ flake (i.e., its number of layers, *N*), it is first necessary to specify some parameters that can have a direct influence on the quality and accuracy of the results. The first parameter is the wavelength of the incident laser light used in the Raman experiments. As it will be detailed in the following, the measurements of the frequencies, linewidths, and intensity of first-order Raman active phonon modes of MoS_2_ have to be obtained with good accuracy in order to evaluate the thickness of a MoS_2_ flake. These phonons modes are (i) the in-plane phonon mode involving relative motion of Mo and S atoms with E′ symmetry for a monolayer (E^1^_2g_ for bulk) and (ii) the out-of-plane phonon mode involving only out-of-plane motions of S atoms with A′_1_ symmetry for a monolayer (A_1g_ for bulk). These modes are located around 385 and 405 cm^−1^, respectively, in neutral and defect-free MoS_2_ monolayers [33–34]. More precisely, in MoS_2_ multilayers, the symmetries of these phonon modes are E′ and A′_1_ for an odd number of layers, and E_g_ and A_1g_ for an even number of layers. For simplicity, hereafter when we will discuss the dependence on *N* of the features of these phonon modes, they will be simply referred to as in the bulk, E^1^_2g_ and A_1g_, independently of the number of layers.

A drastic change of the Raman spectra, especially in the frequency range of the A_1g_ and E^1^_2g_ modes, occurs when the spectra are excited at an energy close to those of the A and B excitons located around 655 nm (1.89 eV) and 601 nm (2.06 eV), respectively, in MoS_2_ monolayers [35–36]. When the incident laser energy is in the range of the A and B exciton energies (the so-called resonance conditions), other bands associated to different second-order processes are observed in the Raman spectra with a strong intensity, their frequencies, widths, and intensity depending on the excitation energy [36]. In addition, resonance conditions alter the symmetry selection rules of phonons of MoS_2_ [35]. Some of the second-order bands overlap with the A_1g_ and E^1^_2g_ modes, complicating the exact determination of the parameters of these modes recorded under resonance conditions. Furthermore, since the MoS_2_ exciton characteristics (energy, width, and spectral weight) can be changed by several factors (e.g., stacking, strain, doping, and defects), the Raman intensities measured with a single laser wavelength close to exciton energies can be affected by external factors and differ for samples elaborated by different methods. For these reasons and in the aim to use Raman spectroscopy to count the number of MoS_2_ layers, one must necessarily work under off-resonance conditions, that is, by using incident laser energy far from both exciton resonance energies. In this work, we chose to perform Raman experiments using 532 nm (2.33 eV) laser excitation, because this is sufficiently far from the energy range of A and B excitons [35].

All Raman spectra reported in this paper were recorded on different samples deposited on SiO_2_/Si(100) substrate. Hence, the second parameter essential to define is the SiO_2_ thickness. Indeed, the multiple reflection interferences that occur in the air/MoS_2_/SiO_2_/Si structure influence significantly the intensity of the phonon modes [28,37]. In this work, we chose to focus on substrates with a SiO_2_ thickness around 90 nm, which corresponds to the first optimum value for MoS_2_ monolayer (*N* = 1) Raman enhancement with a 532 nm excitation energy and also amplifies the signal in the wavelength range of photoluminescence emission (around 650 nm).

The third parameter to define is the power of the 532 nm light, *P*_λ_, impinging the sample. Much of the Raman information available to evaluate the thickness of MoS_2_ flakes is based on the following parameters: (i) on precise measurements of frequency of the A_1g_ and E^1^_2g_ phonon modes of MoS_2_. These lead to a precise knowledge of the frequency difference Δω_A−E_. It was established that Δω_A−E_ depends monotonously on the number of layers, and Δω_A−E_ is largely used as criterion to evaluate the thickness of MoS_2_ flakes [26,29–30]; (ii) on the precise evaluation of the integrated intensities of the phonon modes of MoS_2_, namely *A*(A_1g_) and *A*(E^1^_2g_), with respect to the integrated intensity of the 521 cm^−1^ mode from a bare area of the oxidized silicon substrate, *A*_0_(Si), used as an intensity reference [31], or from the silicon substrate underneath the MoS_2_ flake, *A*_2D_(Si) [28]; (iii) on the precise measurement of the *A*_2D_(Si)/*A*_0_(Si) intensity ratio [31]; and (iv) on the measurement of ultralow-frequency modes, the so-called breathing modes and shear modes. The frequencies and the number of LB and S modes allow one to identify the number of layers [32–33] and the presence of twist between adjacent layers from the vanishing of the S modes in twisted MoS_2_ flakes [20,38–41].

**Figure 1:**
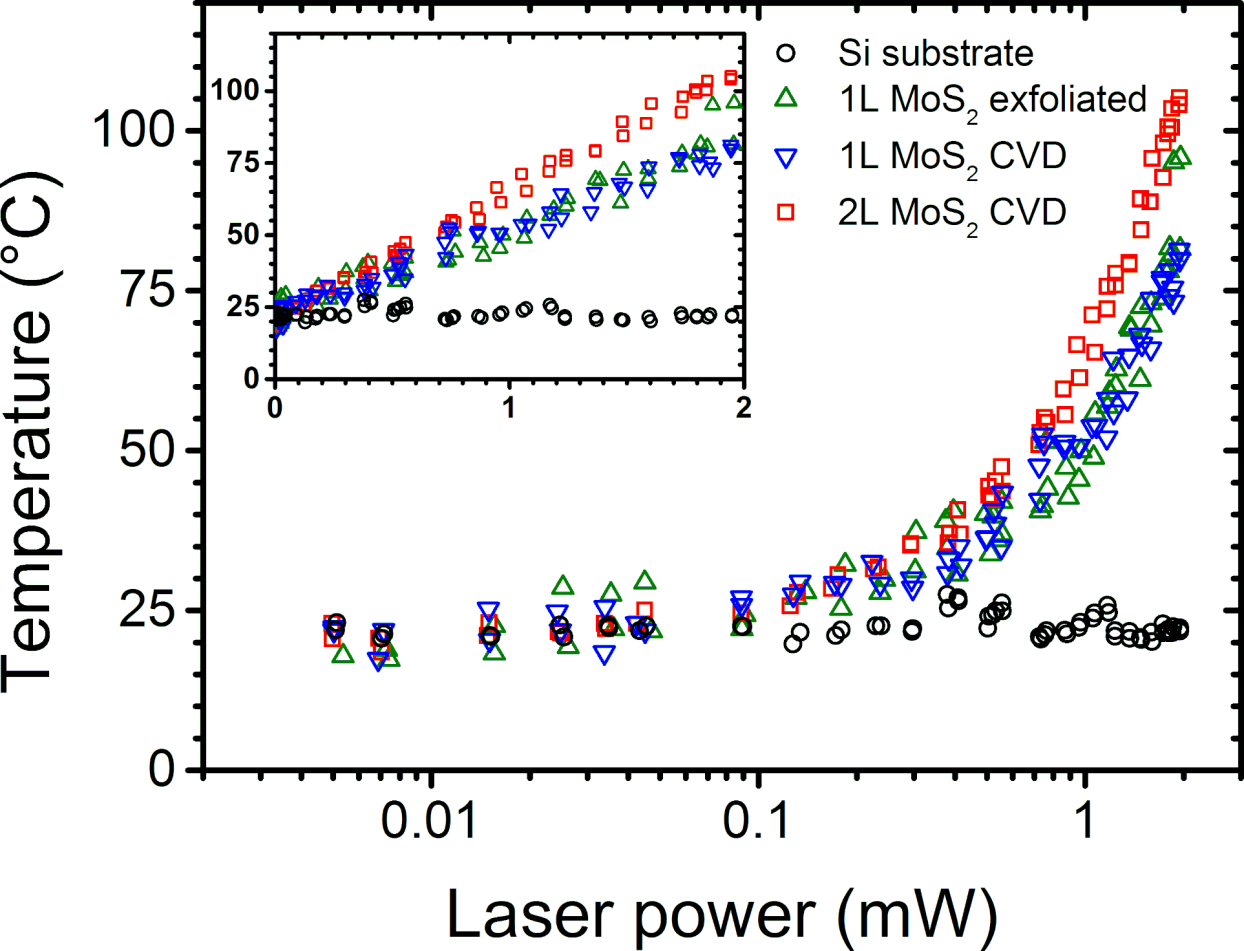
Evolution of the temperature of Si substrate (black circles) and MoS_2_ flakes (1L exfoliated: green upward triangles, 1L CVD: blue downward triangles, and 2L CVD: red squares) as functions of the incident laser power in log-scale. The absolute values of the temperature under the laser spot were measured from the variation of the Stokes/AntiStokes ratio of the 521 cm^−1^ Si mode for the substrate and of the A_1g_ phonon modes for MoS_2_ flakes. Inset, same data plotted with a linear laser power scale.

**Figure 2:**
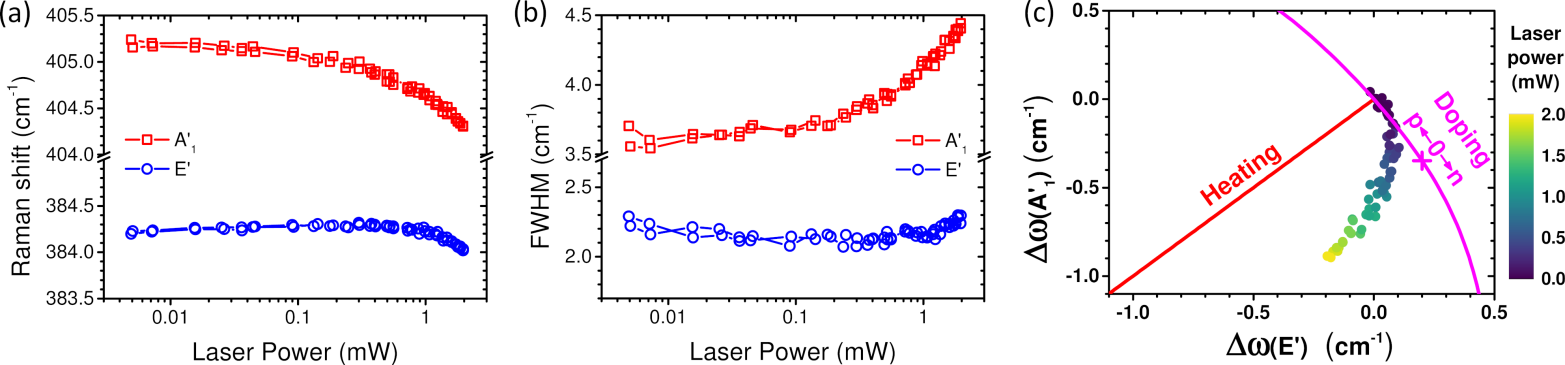
CVD 1L-MoS_2_. Evolution of A′_1_ (red squares) and E′ (blue circles) Raman modes frequencies (a) and full width at half maximum (b) as functions of the incident laser power during a cycle from 5 μW up to 2 mW and back to 5 μW. (c) Evolution of the A′_1_ relative shift versus the E′ relative shift as a function of laser power. The color code of each point corresponds to the incident laser power as displayed on the color bar. The data are compared to the expected evolutions for heating effect only [43] (red line) and for doping only [44] (magenta line). To enable direct comparison, the strain contribution has been removed, and the corresponding zero doping point is labeled as well as the directions corresponding to *p* and *n* doping.

Then, it is essential to determine the limit value of the laser power so that the above measurements are not affected by laser irradiation. Figure 1 shows the evaluation of the temperature of MoS_2_ flakes prepared in different ways and that of the Si substrate as functions of the laser power impinging on the sample through a 100× objective (N.A. 0.9). The power was cycled between ≈5 μW and ≈2 mW. The temperature of MoS_2_ flakes is evaluated from the Stokes/anti-Stokes intensity ratio of A_1g_ phonon modes (similar results are obtained using E^1^_2g_) and that of silicon from the Stokes/anti-Stokes intensity ratio of the 521 cm^−1^ Si mode (see [42] for method details). While the silicon temperature is quasi-insensitive to *P*_λ_, the temperature of MoS_2_ flakes changes monotonically, reversibly, and quasi-linearly with *P*_λ_ (see inset of Figure 1). For MoS_2_, we found an increase rate of 25–30 °C/mW for monolayers (1L-MoS_2_) and 40–45 °C/mW for bilayers (2L-MoS_2_). Usual effects of sample heating are the frequency shift of the phonon modes and their concomitant broadening. In Supporting Information File 1, the frequency and the linewidth of the Si mode as functions of the laser power are displayed (Figure S2). These two parameters are found to be insensitive to *P*_λ_ below 0.5 mW. More intriguing is the evolution of the frequency (Figure 2a) and width (Figure 2b) of the phonon modes of 1L-MoS_2_ as a function of *P*_λ_. For a simple thermal effect [43] and given the 25–30 °C/mW temperature increase rate determined previously, the frequencies of A′_1_ and E′ modes should both downshift by 0.3–0.4 cm^−1^/mW and the width of A′_1_ should increase by ≈0.2 cm^−1^/mW (the width of E′ should remain constant) contrary to what is observed in Figure 2a,b. To clarify this point, we present in Figure 2c (filled dots) the relative shift of the frequency of the A′_1_ mode versus that of the E′ mode measured on CVD 1L-MoS_2_ at different laser powers. In the same plot the expected shifts of these modes are reported (i) as functions of a pure thermal effect (Figure 2c, red line) [43] and (ii) as functions of the doping state (Figure 2c, magenta curve) [44]. Clearly the relative shift of the A′_1_ mode frequency versus that of the E′ mode frequency as a function of *P*_λ_ significantly differs from the behavior expected by considering a simple thermal effect. Consequently, the results reported in Figure 2c clearly evidence photo-doping of 1L-MoS_2_ concomitant with a thermal effect, as already observed for MoS_2_ on SiO_2_/Si [45] as well as for graphene [42]. Furthermore, the evolution of the A′_1_ and E′ widths with *P*_λ_ (Figure 2b), that is, the weak change of the E′ width and the significant increase of the A′_1_ width concomitant with the A′_1_ frequency decrease, support this interpretation [44]. For a laser power smaller than 0.3 mW, photo-doping remains rather low, but it is the dominant contribution to the shift of the modes. Similar results were obtained on other samples including exfoliated 1L-MoS_2_. The effects were found irreversible in some cases when *P*_λ_ exceeded 1 mW but remained always reversible if *P*_λ_ was kept below 1 mW.

Based on the above information, all Raman results reported and discussed in this paper were obtained by using a *P*_λ_ around 0.1 mW chosen as a good compromise between mitigating laser effects and maintaining measurement efficiency to ensure the accuracy of the Raman criteria discussed in the next part of this paper.

In summary, unless specified otherwise, all Raman spectra reported and discussed in this paper were recorded at an excitation wavelength of 532 nm, with a laser working-power close to 0.1 mW, and using a 100× objective (N.A. 0.9), on MoS_2_ flakes or thin films deposited on SiO_2_/Si substrate with a SiO_2_ thickness of 90 ± 6 nm.

### Application of Raman criteria to characterize MoS_2_ flakes

In this part, we report and discuss the advantages and limits of some Raman criteria that were found to be efficient to derive the thickness (i.e., the number of layers *N*) of large MoS_2_ flakes prepared by different ways, namely mechanical exfoliation and standard CVD (including twisted CVD 2L-MoS_2_). Then, we discuss the application of Raman spectroscopy to characterize samples synthesized by DLI-PP-CVD. In contrast to the first two kinds of MoS_2_ samples, the latter are constituted of nanoflakes with possibly a distribution of thicknesses and twist angles between adjacent layers of multilayer domains as well as a higher number of defects.

#### Exfoliated MoS_2_ flakes as reference samples

We performed Raman experiments on mechanically exfoliated MoS_2_ [1] that will serve as reference samples. The stacking sequence in exfoliated MoS_2_ flakes is of the 2Hc-type [34]. The common feature of all these samples is to have a limited number of defects. Note also that all exfoliated flakes have a lateral size (few micrometers at minimum) significantly larger than the diameter of the laser spot. In such flakes, the exact number of layers, *N*, is determined by combining optical microscopy, spectral reflectivity, and the measurement of the breathing modes and shear modes in the ultralow frequency (ULF) range of the spectra [32–34].

**Figure 3:**
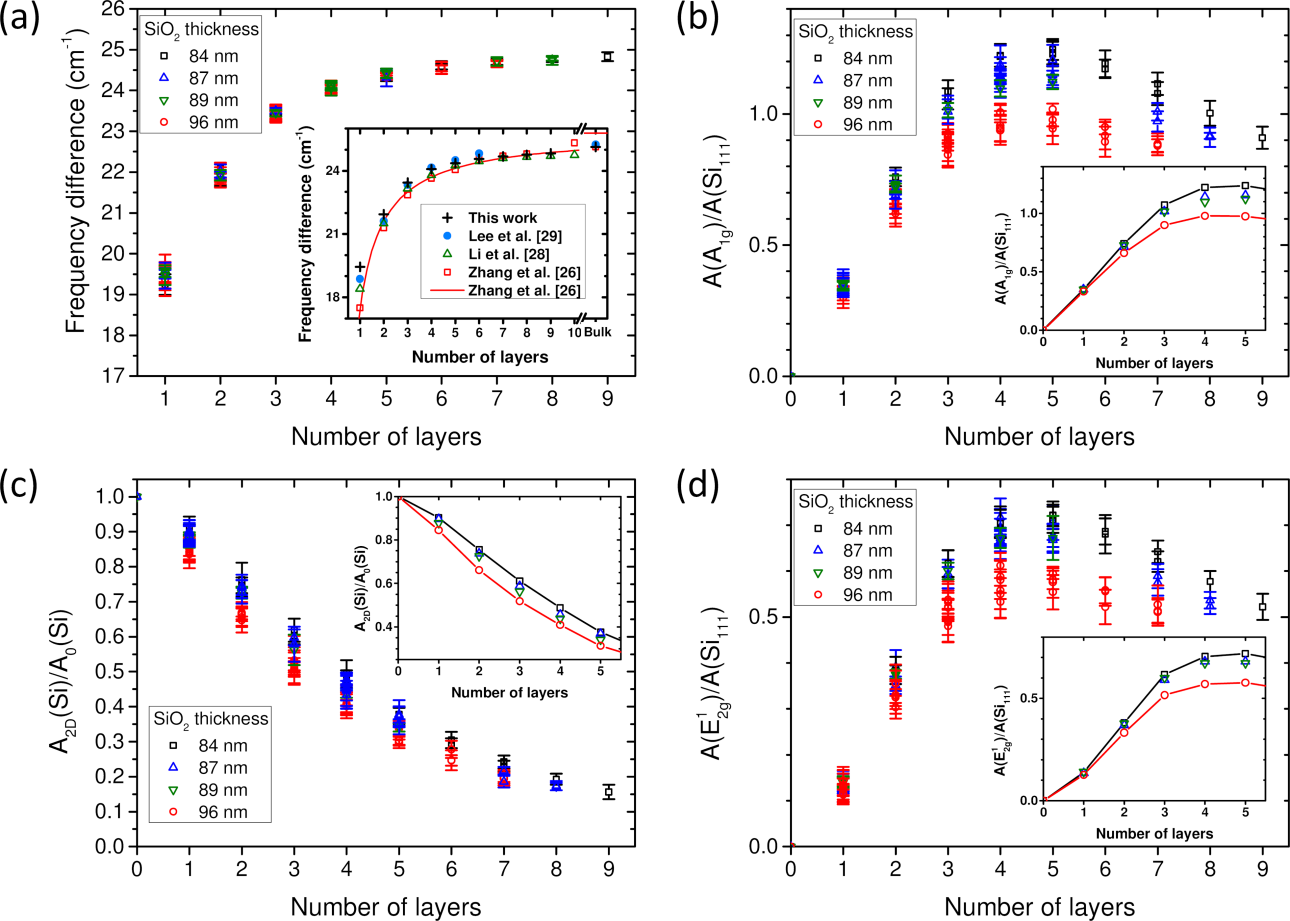
Mechanically exfoliated samples on SiO_2_/Si substrates with four different SiO_2_ thicknesses specified in the legends. Dependence on the number of MoS_2_ layers (a) of the frequency difference between the A_1g_ and E^1^_2g_ phonon modes, and of the normalized integrated intensities (see text) of (b) the A_1g_, (c) the Si 521 cm^−1^, and (d) the E^1^_2g_ modes. The inset in (a) shows a comparison of the average values measured here with data from the literature. The insets in (b–d) show the corresponding average values for each SiO_2_ thickness and each number of layers.

One of the most popular criteria to determine the number of layers of MoS_2_ flakes is the measurement of Δω_A−E_, that is, the frequency difference between the A_1g_ and E^1^_2g_ phonon modes [26,29–30]. Figure 3a shows the dependence of Δω_A−E_ on the number of layers measured on exfoliated MoS_2_ flakes deposited on Si/SiO_2_ substrates with four different SiO_2_ thicknesses. As previously well documented in the literature, we confirm that Δω_A−E_ depends monotonously on the number of layers and does not depend on the SiO_2_ thickness (Figure 3a). The separation between *N* and *N* + 1 values are larger than the experimental uncertainties (error bars in the graph) up to *N* = 3. The error bars start to overlap between *N* = 4 and *N* = 5. Comparison with data from the literature (see inset in Figure 3a) shows that this overlap occurs even between *N* = 3 and *N* = 4 when additional variability due to setup and samples is taken into account. Above *N* = 4, the separation becomes too small compared to the uncertainty. Thus, the measurement of Δω_A−E_ in exfoliated MoS_2_ flakes allows one to evaluate with good accuracy the number of layers for *N* ≤ 3. It is then necessary to supplement the Δω_A−E_ criterion with others to reliably count thick multilayers. In addition, we will establish in the following that this criterion has to be taken with care to derive *N* in MoS_2_ samples other than reference exfoliated MoS_2_, because the A_1g_ and E^1^_2g_ frequencies, and thus the value of Δω_A−E_, can be affected by different factors such as stacking order, strain, doping, and defects which can be present in MoS_2_ flakes prepared by other ways [44,46–49].

To evaluate the number of layers, we can also use information associated with the integrated intensity of MoS_2_ phonon modes. Figure 3b and Figure 3d show, respectively, the dependences of the normalized integrated intensities of the A_1g_ and the E^1^_2g_ mode as functions of *N* for four values of the SiO_2_ thickness. For normalization, we use here an external reference, which is a bare Si(111) wafer with only native oxide. In the following, *A*(Si_111_) stands for the integrated intensity of the Si(111) 521 cm^−1^ mode. This reference is preferred to the Si(100) substrate with 90 ± 6 nm SiO_2_ to avoid the effects of the SiO_2_ thickness variations and crystal orientation. For comparison with other setups or references, the polarization ratio of our setup and the relative values measured on Si(100) with native oxide and 90 nm SiO_2_ are given in Supporting Information File 1. As noted by several authors and predicted by the optical interference model, the normalized integrated intensities of MoS_2_ modes, namely *A*(A_1g_)/*A*(Si_111_) and *A*(E^1^_2g_)/*A*(Si_111_), increase first with *N* and then decrease showing a maximum for *N* = 4–5 for all SiO_2_ thicknesses. Obviously, this non-monotonous dependence prevents using these measurands alone to evaluate the number of layers for *N* > 4. Moreover, a significant dependence of the MoS_2_ Raman intensity on the SiO_2_ thickness occurs for *N* > 2, pointing out the importance to determine precisely this latter parameter.

Another criterion to derive the thickness of MoS_2_ flakes is the *A*_2D_(Si)/*A*_0_(Si) intensity ratio [31]. For the evaluation of this ratio, it is of great practical advantage to use the same silicon (the silicon below the oxide, which is Si(100) in the present work) in the measurement of *A*_2D_(Si) and *A*_0_(Si). A necessary precaution is that the Si(100) substrate orientation has to be kept the same for both measurements. Another advantage is to give a common origin to the plots of *A*_2D_(Si)/*A*_0_(Si) as a function of *N* (*A*_2D_(Si)/*A*_0_(Si) = 1 for *N* = 0) for any SiO_2_ thickness.

Figure 3c displays the *A*_2D_(Si)/*A*_0_(Si) ratio measured on exfoliated MoS_2_ flakes deposited on SiO_2_/Si(100) substrates with four different SiO_2_ thicknesses as a function of *N*. We confirm the monotonous decrease of the *A*_2D_(Si)/*A*_0_(Si) ratio with increasing *N* [31]. For each *N*, the value of this ratio depends on the SiO_2_ thickness (Figure 3c; black, blue, green, and red symbols correspond to a SiO_2_ thickness of 84, 87, 89, and 96 nm, respectively). Despite the monotonous dependence of the *A*_2D_(Si)/*A*_0_(Si) ratio, its dependence on SiO_2_ thickness combined with experimental errors lead to the conclusion that the measured values for *N* and *N* + 1 can overlap for any *N* if the SiO_2_ thickness is not known with good accuracy. For a given SiO_2_ thickness, the gap between the *A*_2D_(Si)/*A*_0_(Si) ratio for *N* and *N* + 1 is sufficient to ensure a rather good reliability only for *N* ≤ 5.

In summary, for exfoliated MoS_2_, considering jointly the three Raman criteria (i) value of Δω_A−E_, (ii) value of the normalized integrated intensities of the A_1g_ and E^1^_2g_ modes, and (iii) value of the *A*_2D_(Si)/*A*_0_(Si) ratio, one can unambiguously derive the number of layers as long as *N* ≤ 4 and the SiO_2_ thickness is precisely known. It has also been suggested in the literature to use the intensity ratio *A*(A_1g_)/*A*_2D_(Si) (or equivalently *A*(E^1^_2g_)/*A*_2D_(Si)). As it will be discussed in the following, we see two major problems with this approach. The first is the dependence of the Si signal on the crystal orientation and the SiO_2_ thickness. The second relates to the fact that using this ratio, rather than using each measurand independently and contrasting them, even if more practical, can hide some information.

Finally, we compare in Supporting Information File 1, Figure S3, the dependence on *N* of *A*(A_1g_)/*A*(Si_111_) and *A*_2D_(Si)/*A*_0_(Si) for three SiO_2_ thicknesses and two microscope objectives with different numerical apertures, N.A. = 0.9 (blue symbols in Figure S3), and N.A. = 0.5 (red symbols in Figure S3). We observe that the normalized integrated intensity of the A_1g_ mode, *A*(A_1g_)/*A*(Si_111_) (Figure S3a–c), is independent of the value of N.A. Concerning the dependence on *N* of *A*_2D_(Si)/*A*_0_(Si) (Figure S3d–f), we found that this ratio is slightly smaller for N.A. = 0.5 than for N.A. = 0.9 and is in a better agreement with the model of Li and coworkers [31] (black solid line in Figure S3d–f). (Note that in this latter work the experimental data on which the model has been adjusted were recorded using a numerical aperture N.A. ≈ 0.45).

#### MoS_2_ flakes prepared by CVD

In this part, we analyze the pertinence of the previous criteria to derive the thickness of large MoS_2_ flakes synthesized by CVD. In a first part, we probe the effectiveness of these criteria to evaluate the thickness of large standard CVD MoS_2_ flakes. Such flakes have a limited number of defects and, like in exfoliated MoS_2_, the stacking sequence is of the 2Hc type. In the second part, we examine the relevance of these criteria to evaluate the thickness of twisted CVD MoS_2_ flakes.

**Standard CVD MoS_2_ flakes:** As derived from the features of the LB and S ultralow-frequency modes, these samples do not show any twist between adjacent layers (presence of S modes for all flakes with *N* ≥ 2). The flakes are thus characterized by 2Hc stacking (or close to 2Hc stacking) and a low number of defects, and are named standard CVD MoS_2_ flakes. On the basis of the latter features, the structure of these flakes is close to that of exfoliated MoS_2_ flakes. However, the high temperature used in the CVD synthesis and interaction with the substrate can lead to lattice distortion and the presence of vacancies and doping. In the following, we limit our study to a number of layers *N* ≤ 4.

**Figure 4:**
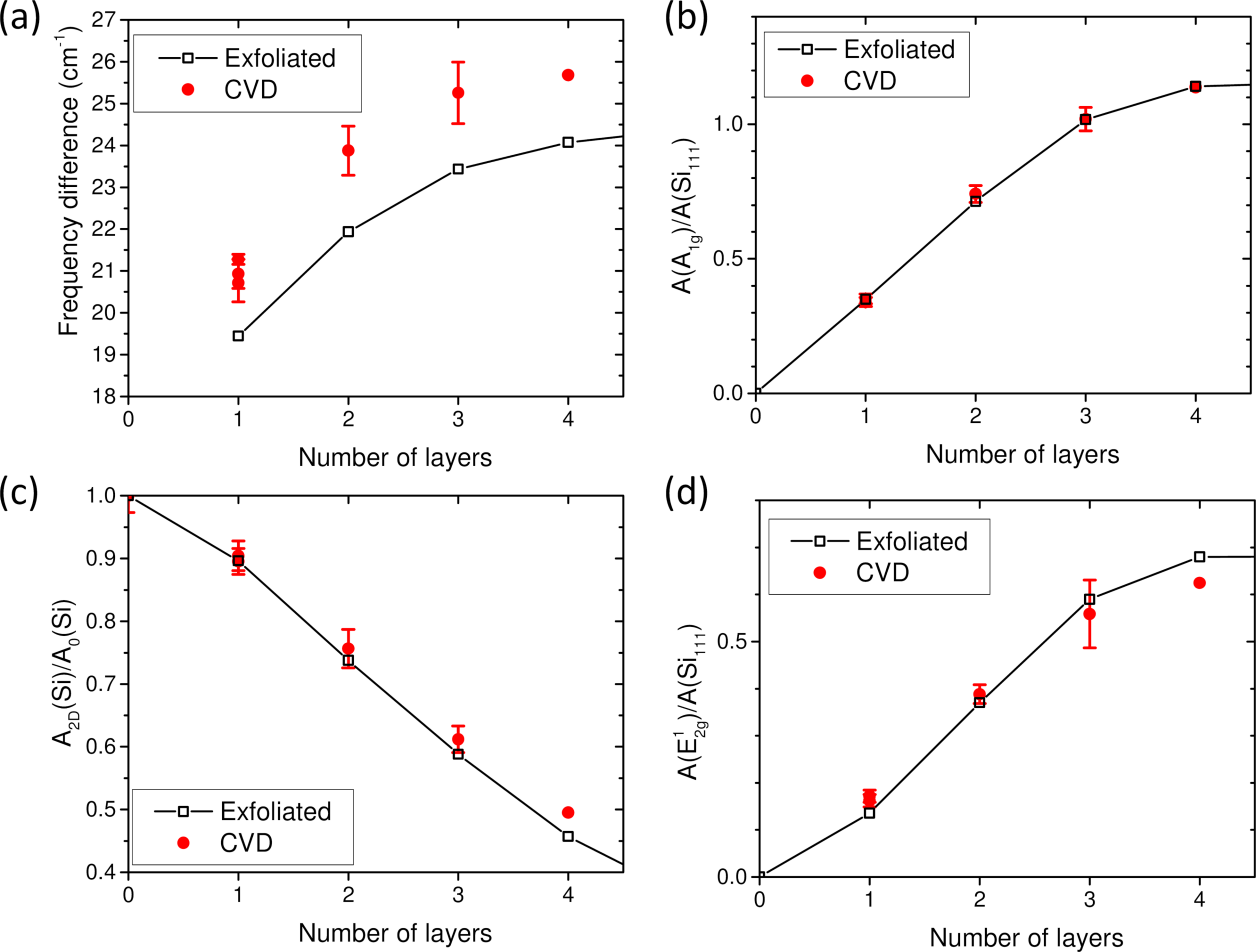
Comparison between CVD (red filled dots) and mechanically exfoliated (black-line-connected open squares) MoS_2_ on a 87 nm SiO_2_/Si substrate. Dependence on the number of MoS_2_ layers of (a) the frequency difference between the A_1g_ and E^1^_2g_ phonon modes, and of the normalized integrated intensities (see text) of (b) the A_1g_, (c) the Si 521 cm^−1^, and (d) the E^1^_2g_ modes. For the 4L CVD, only few points are measured and the errors are not shown because they cannot be properly derived.

Figure 4a compares the values of Δω_A−E_ measured on exfoliated (Figure 4a, black symbols) and standard CVD MoS_2_ flakes (Figure 4a, red symbols) for *N* ≤ 4. As previously, the exact number of layers is obtained by combining optical microscopy, spectral reflectivity, and number and frequencies of LB and S modes. For both kinds of MoS_2_ flakes, Δω_A−E_ increases monotonously with *N*, but for the same *N,* the values of Δω_A−E_ are systematically larger in standard CVD MoS_2_ flakes. We attribute this discrepancy mainly to a difference of strain states between the two kinds of samples. Exfoliated samples are mostly found with low or slight compressive strain, while CVD samples are under tension. Other factors such as doping, defects, or stacking were shown to lead to large changes of Δω_A−E_ [44,46,49]. In summary, the value of Δω_A−E_ is clearly and significantly sample-dependent. Consequently, Δω_A−E_ cannot be considered as a definitive criterion to derive the number of layers in any of MoS_2_ flakes prepared in different ways. In other words, one cannot define a single master curve, Δω_A−E_ vs *N*, which would be valid for all the MoS_2_ flakes independently of their preparation method or environment.

The dependencies on *N* of the normalized integrated intensities of A_1g_ and E^1^_2g_ modes and the *A*_2D_(Si)/*A*_0_(Si) ratio measured on standard CVD flakes are compared with the average values of exfoliated samples with the same substrate SiO_2_ thickness (Figure 4b–d). In contrast to Δω_A−E_, the *N* dependencies of these intensities are very close in exfoliated and standard CVD MoS_2_ flakes. Only *A*_2D_(Si)/*A*_0_(Si) and the normalized integrated intensity of the E^1^_2g_ phonon modes slightly differ for *N* = 4. However, this may be due to the fact that the statistics is rather poor on this measurement, because this flake is rather small compared to the others. With regards to these results, these measurands give important information to evaluate the number of layers of 2Hc-stacked MoS_2_ flakes independently of the elaboration procedure as long as *N* ≤ 4.

**Twisted CVD MoS_2_ flakes:** Other interesting samples are large CVD MoS_2_ flakes that present a twist angle, θ, between adjacent layers. We exemplify here the complexity to characterize such samples from the previous Raman criteria with the case of twisted MoS_2_ bilayers. The identification of the bilayer character of the investigated flakes was unambiguously obtained independently from spectral reflectivity and optical contrast.

**Figure 5:**
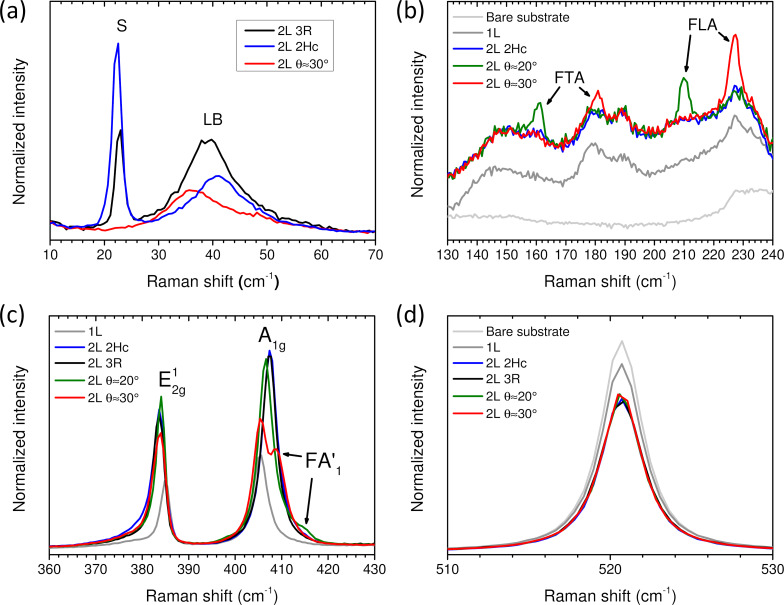
θ-2L-MoS_2_. (a) Raman spectra of 3R-, 2Hc- and 30°-2L-MoS_2_ (black, blue, and red lines, respectively) in the ultralow-frequency region. Layer breathing (LB) and shear (S) modes are labelled. (b) Raman spectra of 2Hc-, 20°- and 30°-2L-MoS_2_, 1L-MoS_2_, and bare substrate in the region of 130–240 cm^−1^. Raman modes with θ-dependent frequencies (FTA and FLA) are labelled. (c) Raman spectra of 2Hc-, 3R-, 20°- and 30°-2L-MoS_2_, and 1L-MoS_2_ in the region of 360–430 cm^−1^. E^1^_2g_, A_1g_, and θ-dependent FA′_1_ are labelled. (d) Raman spectra of 2Hc-, 3R-, 20°- and 30°-2L-MoS_2_, 1L-MoS_2_, and bare substrate in the region of the Si 521 cm^−1^ mode. Y-scale intensities are normalized to the incident laser power and the acquisition time. Presented spectra are averages extracted from Raman maps.

Figure 5a shows the low-frequency range of spectra recorded on three types of MoS_2_ bilayer (named 2L-MoS_2_ in the following), namely a bilayer with θ ≈ 30° (this sample belongs to the so-called twisted-bilayer family for which 0 < θ < 60° and is named in the following as θ-2L-MoS_2_), the so-called 2Hc-2L-MoS_2_ and the so-called 3R-2L-MoS_2_. In the latter structure, the stacking between two adjacent layers corresponds to a twist angle of θ = 0°, and it is such that the S atoms of the top monolayer are superimposed on the Mo atoms of the bottom monolayer, and the Mo atoms of the top monolayer are above the hexagon centers of the bottom monolayer [50]. The spectrum in the low-frequency range is dominated by the contributions of the LB and S modes, the frequencies of these modes depending on the twist angle [20,39]. The LB mode emerges in the Raman spectra of all 2L-MoS_2_ samples. In line with previous results [20], the Raman shift of the peak position of the LB mode in 3R-2L-MoS_2_ is smaller than that of the 2Hc-2L-MoS_2_, and the LB mode Raman shift in 30°-2L-MoS_2_ is even smaller. Also, in agreement with the literature [20,38–41], the S mode vanishes in 30°-2L-MoS_2_.

**Figure 6:**
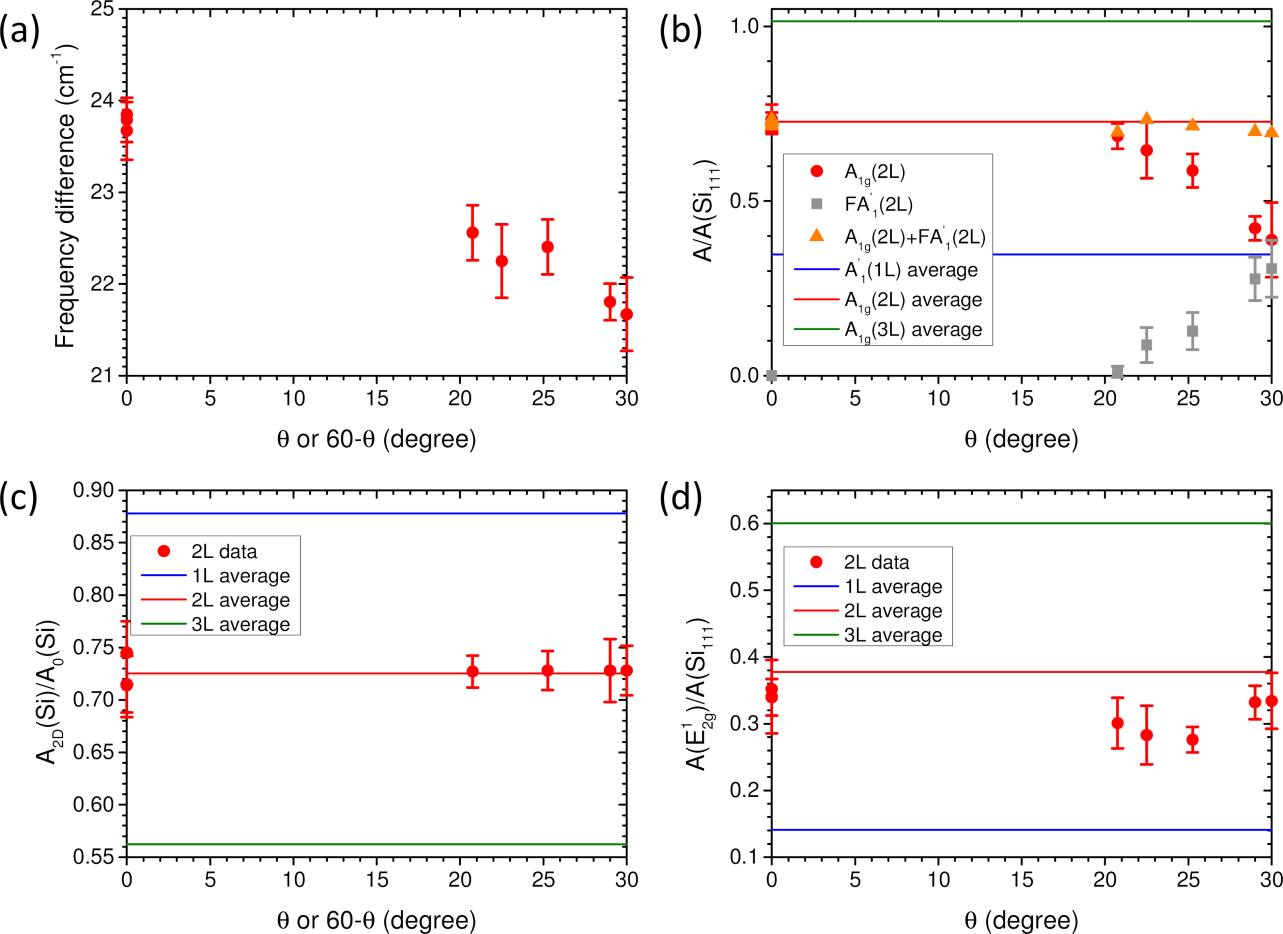
θ-2L-MoS_2_. θ-dependence of the frequency difference between the A_1g_ and E^1^_2g_ phonon modes (a); θ-dependence of the normalized integrated intensities (see text) of A_1g_ and FA′_1_ modes and their sum (b), of the Si 521 cm^−1^ mode (c), and of the E^1^_2g_ mode (d). In (b–d), the corresponding average values for 1L-MoS_2_, 2Hc 2L-MoS_2_, and 3L-MoS_2_ are plotted as horizontal lines (blue, red, and green, respectively) for comparison.

In Figure 5b, the 130–240 cm^−1^ range of the Raman spectra recorded on monolayer, 2Hc-2L-MoS_2_, and two θ-2L-MoS_2_ is displayed. As well documented in the literature, this frequency range is dominated by the contributions of second-order Raman processes [20,26]. The general profile of the spectra is similar in 1L-MoS_2_, 2Hc-2L-MoS_2_, and θ-2L-MoS_2_ with the exception that in the latter flakes, new bands, named FLA and FTA, are superimposed to the second-order Raman spectra. The FLA and FTA modes in θ-2L-MoS_2_ are attributed, respectively, to folded longitudinal acoustic phonons and folded transverse acoustic phonons of the monolayer due to the presence of a moiré superlattice [20]. As shown in the literature [20], the frequencies of these modes depend on the twist angle (see Figure 2e in [20]). Unfortunately, these dependencies show a mirror behavior with respect to θ = 30°. This means that from given FLA and FTA positions, two values are possible: θ ∈ [0,30]° or its mirror 60° − θ. As a consequence, θ will be given in the range of 0–30° in all plots in Figure 6 with the possibility that the values attributed to θ-2L-MoS_2_ could be 60° − θ instead. For instance, the data from both 2Hc-2L-MoS_2_ and 3R-2L-MoS_2_ are reported at θ = 0° in these plots. From the positions of the FTA and FLA, we claim that the spectra of the two θ-2L-MoS_2_ displayed in Figure 5 correspond to 20°-2L-MoS_2_ (Figure 5b–d, solid green line) and 30°-2L-MoS_2_ (Figure 5a–d, solid red line), respectively.

The dependence of A_1g_ and E^1^_2g_ modes on the twist angle (derived from the positions of FTA and FLA modes) is reported in Figure 5c. The frequency of the E^1^_2g_ mode in 3R-, 2Hc-, and θ-2L-MoS_2_ is downshifted with respect to its frequency in 1L-MoS_2_, and it does not show a clear dependence on the twist angle. In contrast, the profile of the A_1g_ mode significantly depends on the twist angle, and a new mode, named FA′_1_, appears on the high-frequency side of the A_1g_ mode. The FA′_1_ mode is identified as Raman scattering from moiré phonons associated with the A′_1_ dispersion curve of 1L-MoS_2_. It is folded onto the zone center and, consequently, becomes Raman active [20]. Obviously, its frequency depends on the twist angle and the θ-dependence of the FA′_1_ frequency was recently established both theoretically and experimentally (see Figure 3e in [20]). On the basis of these previous results, we have been able to evaluate the value of θ for each 2L-MoS_2_ investigated from the position of the FA′_1_ mode. The values of the angles derived from the position of FTA/FLA and FA′_1_ are in close agreement.

The objective of this work is to characterize the thickness of all MoS_2_ flakes. The relevance of the criteria based on the frequency (Δω_A−E_) and normalized integrated intensity (*A*(A_1g_)/*A*(Si_111_)) of the A_1g_ mode has to be reevaluated in twisted 2L-MoS_2_ flakes. As shown in Figure 5d, the normalized intensity of the 521 cm^−1^ Si mode from the substrate underneath MoS_2_ flakes is close in all the 2L-MoS_2_ and independent of the twist angle.

Figure 6 summarizes and details the dependence on the twist angle of the four Raman criteria defined above for 2L-MoS_2_. In all plots of Figure 6, the values of angles were derived from the positions of FTA, FLA and FA′_1_. The values of the different criteria measured for θ-2L-MoS_2_ are compared with the average values of the same criteria measured on exfoliated 1L-, 2L-, and 3L-MoS_2_ flakes. In θ-2L-MoS_2_, the value of Δω_A−E_ strongly depends on the twist angle and significantly differs from the average value measured in 2Hc-2L-MoS_2_ (Figure 6a). For θ = 30°, the value of Δω_A−E_ is close to the one found in CVD 1L-MoS_2_ [51]. In consequence, using Δω_A−E_ alone could lead to a wrong evaluation of the thickness of twisted 2L-MoS_2_.

The normalized integrated intensity *A*(A_1g_)/*A*(Si_111_) significantly decreases when the twist angle increases (Figure 6b, red dots), and in 30°-2L-MoS_2_, the value of *A*(A_1g_)/*A*(Si_111_) is close to the average value found in 1L-MoS_2_ (Figure 6b, blue solid line). The behavior of *A*(A_1g_)/*A*(Si_111_) is opposite to the one of the normalized integrated intensity of the FA′_1_ mode, *A*(FA′_1_)/*A*(Si_111_), the latter increasing with the twist angle (Figure 6b, gray squares). These results are in qualitative agreement with those reported in [40]. It can be emphasized that the integrated intensity of A_1g_ and FA′_1_ bands taken together (Figure 6b, orange triangles) is close to the average value found for 2Hc-2L-MoS_2_ (Figure 6b, red solid line). The reason for this compensation between *A*(A_1g_) and *A*(FA′_1_) is not clear yet, but it could present a practical advantage in the use of the global integrated intensity of the spectral band, located around the position of the A_1g_ mode for the evaluation of the thickness of twisted MoS_2_ flakes.

We also observed a tendency for *A*(E^1^_2g_)/*A*(Si_111_) to be slightly lower for θ-2L-MoS_2_ than for 2Hc-2L-MoS_2_ (or 3R-2L-MoS_2_, which is similar), but to a lesser extent compared to *A*(A_1g_)/*A*(Si_111_), that is ca. 20% vs ca. 50% at maximum, respectively (Figure 6d). These results are also in qualitative agreement with those reported in [40]. Finally, only the value of the *A*_2D_(Si)/*A*_0_(Si) ratio seems to provide a robust/reliable information to characterize the thickness of MoS_2_ flakes, since it is found largely independent of θ in all measured 2L-MoS_2_ samples (Figure 6c). Even if further work is needed to complete the data presented here with other values of θ and twisted MoS_2_ samples with *N* > 2, we anticipate that the value of *A*_2D_(Si)/*A*_0_(Si) ratio would be close in twisted and 2Hc-stacked MoS_2_ multilayers. However, as previously recalled, the sensitivity of this ratio to the SiO_2_ thickness and the gap between the *A*_2D_(Si)/*A*_0_(Si) ratios for *N* and *N* + 1 permit to ensure the determination of *N* with a rather good reliability only for *N* ≤ 5.

#### DLI-PP-CVD MoS_2_ nanoflakes

The aim of this part is to define which Raman information is relevant to estimate the thickness of MoS_2_ samples produced by DLI-PP-CVD. These samples are significantly different from the previous ones (exfoliated and standard CVD). Indeed, they are constituted of nanoflakes (with a lateral size of typically 50 nm, i.e., well below the laser spot size) with possibly a distribution of thicknesses and twist angles between adjacent layers of multilayer domains and a higher number of defects (the average inter-defect distance ranges from 3 to 6 nm as estimated from the LA and A_1g_ intensity ratio [52]). In addition, the MoS_2_ surface coverage is a priori unknown and can be incomplete. It is then necessary to implement a first check criterion that ensures that the thickness estimation method based on the comparison with results obtained on exfoliated samples is still valid. More generally, this point is critical for the characterization of samples synthesized using new methods or new precursors that can lead to the co-deposition of several by-products (such as carbon, oxides, and metals), which can significantly change the measured Raman intensities. Based on the results presented in the previous sections, we have shown that the value of the *A*_2D_(Si)/*A*_0_(Si) ratio provides a robust/reliable Raman information to characterize the thickness of MoS_2_ flakes for *N* ≤ 5. However, this parameter does not rely unambiguously on the presence of MoS_2_. The deposition of any other material would influence its value and could lead to a wrong estimation. In the most general case, the sample characteristics are not perfectly known and can be significantly different from the reference characteristics. As a consequence, it seems mandatory to compare the thickness estimated from the *A*_2D_(Si)/*A*_0_(Si) ratio with other measurands directly related to the presence of MoS_2_. To this aim, we propose to use jointly the normalized integrated intensity of the MoS_2_ phonon modes, namely *A*(A_1g_)/*A*(Si_111_) and/or *A*(E^1^_2g_)/*A*(Si_111_).

**Figure 7:**
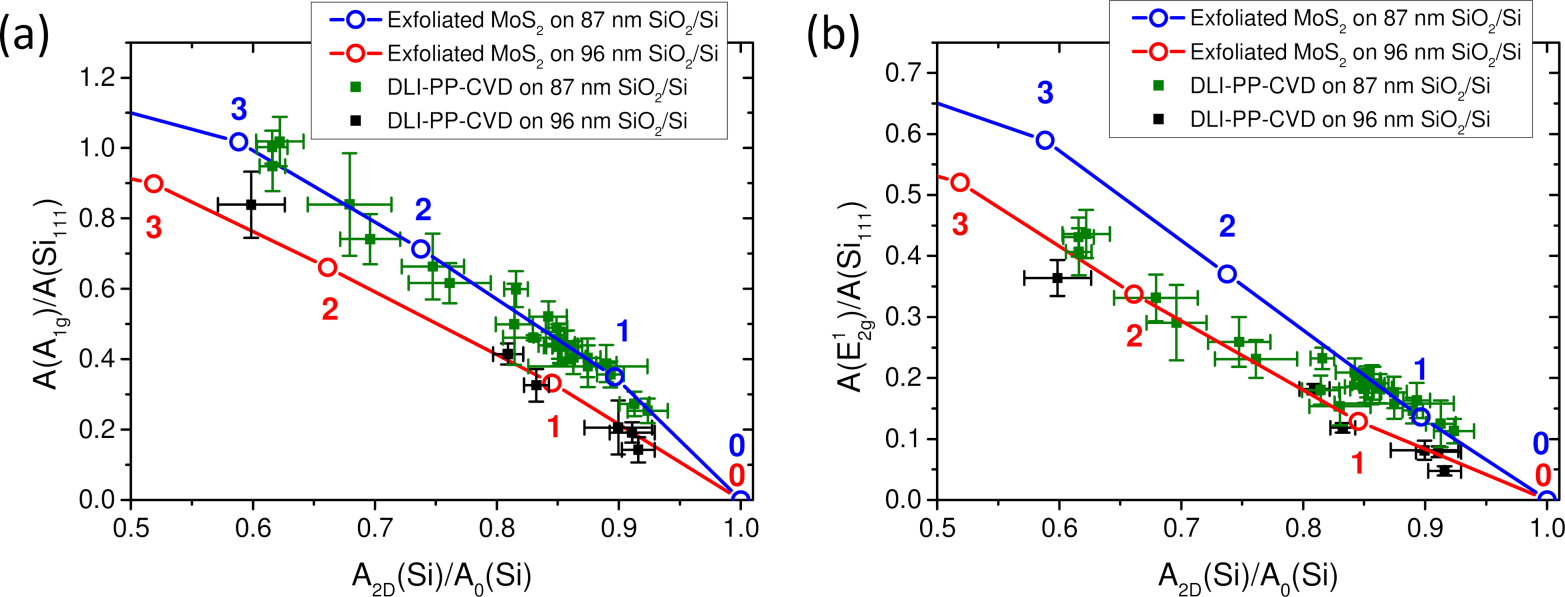
DLI-PP-CVD samples. Normalized integrated intensities of (a) the A_1g_ and (b) the E^1^_2g_ modes as functions of the normalized integrated intensities of the Si 521 cm^−1^ mode of DLI-PP-CVD samples on 87 nm (green squares) and 96 nm (black squares) SiO_2_ on Si substrates. Each point corresponds to the average value extracted from 121-point Raman maps. The average values measured on corresponding exfoliated MoS_2_ samples are also plotted for comparison as open dots (blue and red, respectively, for 87 nm and 96 nm SiO_2_), and the corresponding number of layers are provided. The lines are guides to the eye.

In Figure 7, the values of *A*(A_1g_)/*A*(Si_111_) (Figure 7a) and *A*(E^1^_2g_)/*A*(Si_111_) (Figure 7b) are plotted as functions of the value of *A*_2D_(Si)/*A*_0_(Si). In these graphs, the data obtained on DLI-PP-CVD samples are compared with the average reference measurements established previously on exfoliated MoS_2_ deposited on Si/SiO_2_ substrate with the same SiO_2_ thicknesses, namely 96 nm (red open dots in Figure 7) and 87 nm (blue open dots in Figure 7). Note that in the exfoliated samples, the exact number of layers *N* is perfectly known and given on the plots of Figure 7 close to corresponding open dots. The idea behind this representation comes from the expectation that the presence of contaminations or deposition of others species would have a different impact on the Raman intensity coming from MoS_2_ in the film and on the one coming from the Si substrate underneath the deposited thin film. It is, thus, expected that the measurements on contaminated or highly defective MoS_2_ thin films will fall off the reference curve. Indeed, data obtained on poorly crystalline MoS_2_ films synthesized by DLI atomic layer deposition (not shown) are found systematically and significantly below the corresponding reference curve. Concerning the DLI-PP-CVD samples presented in Figure 7, the *A*(A_1g_)/*A*(Si_111_) vs *A*_2D_(Si)/*A*_0_(Si) dependence is found fully compatible with the respective reference exfoliated curves (Figure 7a). The *A*(E^1^_2g_)/*A*(Si_111_) vs *A*_2D_(Si)/*A*_0_(Si) data points mainly agree for thin layers (*A*_2D_(Si)/*A*_0_(Si) > 0.8) but fall systematically below the corresponding reference exfoliated curves for thicker layers (0.8 > *A*_2D_(Si)/*A*_0_(Si) > 0.6) as shown in Figure 7b.

**Figure 8:**
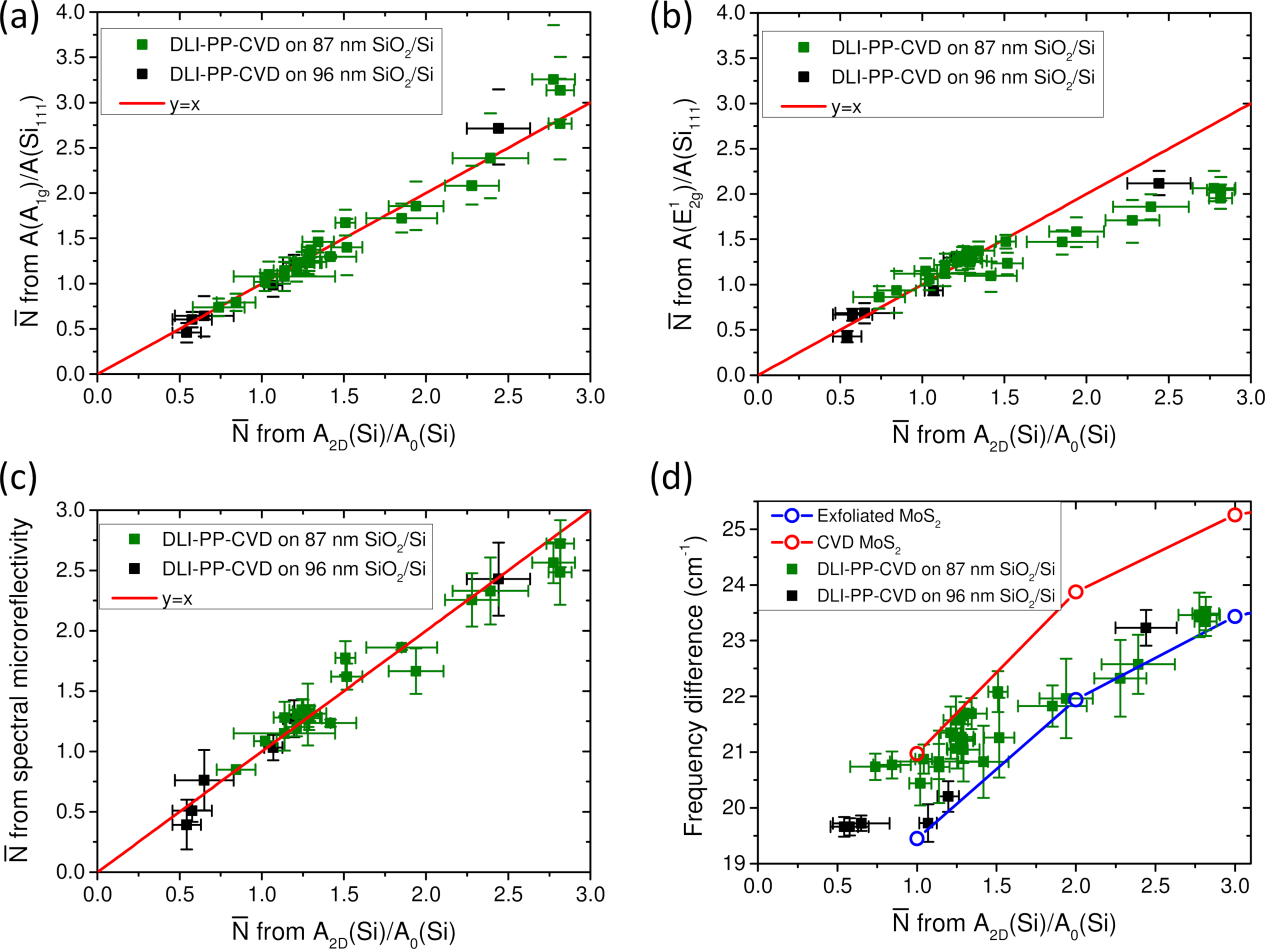
DLI-PP-CVD samples. Average number of layers of DLI-PP-CVD samples obtained by interpolation from exfoliated data of normalized integrated intensities of (a) the A_1g_ and (b) the E^1^_2g_ modes, and of (c) microreflectivity spectra as functions of the average number of layers obtained by interpolation from exfoliated data of normalized integrated intensities of the Si 521 cm^−1^ mode. The red lines in (a–c) correspond to *y* = *x*. (d) Frequency difference between the A_1g_ and E^1^_2g_ phonon modes of DLI-PP-CVD samples as a function of the average number of layers obtained by interpolation from exfoliated data of normalized integrated intensities of the Si 521 cm^−1^ mode. Green (respectively black) squares correspond to DLI-PP-CVD samples synthesized on 87 nm (respectively 96 nm) SiO_2_/Si substrates. In (d), the average values measured on corresponding exfoliated (respectively CVD) MoS_2_ samples are also plotted for comparison as open blue (respectively red) dots. The lines are guides to the eye.

Another way to compare the results is estimating the thickness of DLI-PP-CVD samples by interpolation from exfoliated data of the measured values for *A*_2D_(Si)/*A*_0_(Si), *A*(A_1g_)/*A*(Si_111_), and *A*(E^1^_2g_)/*A*(Si_111_). In Figure 8a (respectively 8b), the average number of layers (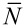) obtained using *A*(A_1g_)/*A*(Si_111_) (respectively *A*(E^1^_2g_)/*A*(Si_111_)) are plotted as a function of the number derived from *A*_2D_(Si)/*A*_0_(Si). It can be emphasized that non-integer values are found for 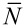, indicating the presence of a mix with unknown proportions of bare substrate (0L), 1L-MoS_2_, 2L-MoS_2_, 3L-MoS_2_, and so on in the investigated DLI-PP-CVD films. It is also noticeable that the errors of 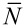 estimated from *A*(A_1g_)/*A*(Si_111_) become larger when 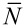 is close to 3 as a consequence of the smoother dependence of this parameter with 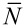. In agreement with the conclusion drawn above from Figure 7, Figure 8a illustrates the coherence between the values of 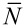 derived from *A*(A_1g_)/*A*(Si_111_) and *A*_2D_(Si)/*A*_0_(Si). All data remain close to the red solid line that represents the ideal relation *y*[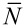 via *A*(A_1g_)/*A*(Si_111_)] = *x*[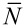 via *A*_2D_(Si)/*A*_0_(Si)]. Figure 8b as well confirms that the values of 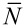 derived from *A*(E^1^_2g_)/*A*(Si_111_) and *A*_2D_(Si)/*A*_0_(Si) agree well for 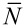 < 1.5, but the values of 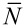 from *A*(E^1^_2g_)/*A*(Si_111_) are systematically lower than those obtained from *A*_2D_(Si)/*A*_0_(Si) when 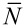 > 1.5. One explanation could the presence of a larger proportion of multilayer regions in the thicker samples, for which, if they are twisted, *A*(E^1^_2g_)/*A*(Si_111_) has been shown to be attenuated in the previous section. If so, the question then arises why the same behavior is not observed for *A*(A_1g_)/*A*(Si_111_) contrary to what would be expected. A possibility could be that because of the observed broadening of the A_1g_ mode in DLI-PP-CVD samples (presumably due to local heterogeneities in terms of doping, strain, defects, or thickness), the FA′_1_ mode becomes indistinguishable from the A_1g_ mode. As a consequence, the intensity of FA′_1_ would merge with *A*(A_1g_) and compensate its attenuation. Other explanations relying on the presence of defects or strain cannot be disregarded, and further works are needed to fully clarify this point.

In order to further confirm the validity of the estimations of 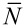 for DLI-PP-CVD samples, we compare in Figure 8c the 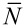 values derived from *A*_2D_(Si)/*A*_0_(Si) with the ones obtained independently from spectral microreflectivity. A very good agreement is found between the two series of data, establishing definitively the relevance of the *A*_2D_(Si)/*A*_0_(Si) ratio to give with good accuracy the average thickness of DLI-PP-CVD MoS_2_ samples for 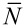 ≤ 3. This agreement justifies the use of the values of 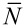 derived from *A*_2D_(Si)/*A*_0_(Si) as abscissa axis in the previous plots.

Finally, in Figure 8d the frequency difference between the A_1g_ and E^1^_2g_ phonons is plotted as a function of 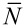 estimated from *A*_2D_(Si)/*A*_0_(Si) for DLI-PP-CVD samples and compared to the data obtained on exfoliated and CVD MoS_2_. DLI-PP-CVD data are distributed between the two curves obtained from the reference samples. This further confirms that this measurand cannot be used to evaluate with good accuracy their average thicknesses. Nevertheless this comparison can be informative, showing that samples with 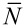 < 1 are most certainly mainly composed of 1L-MoS_2_ and suggesting that the proportions of 2L-MoS_2_, 3L-MoS_2_, or more gradually increase with 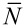, which is compatible with AFM observations (not shown).

**Figure 9:**
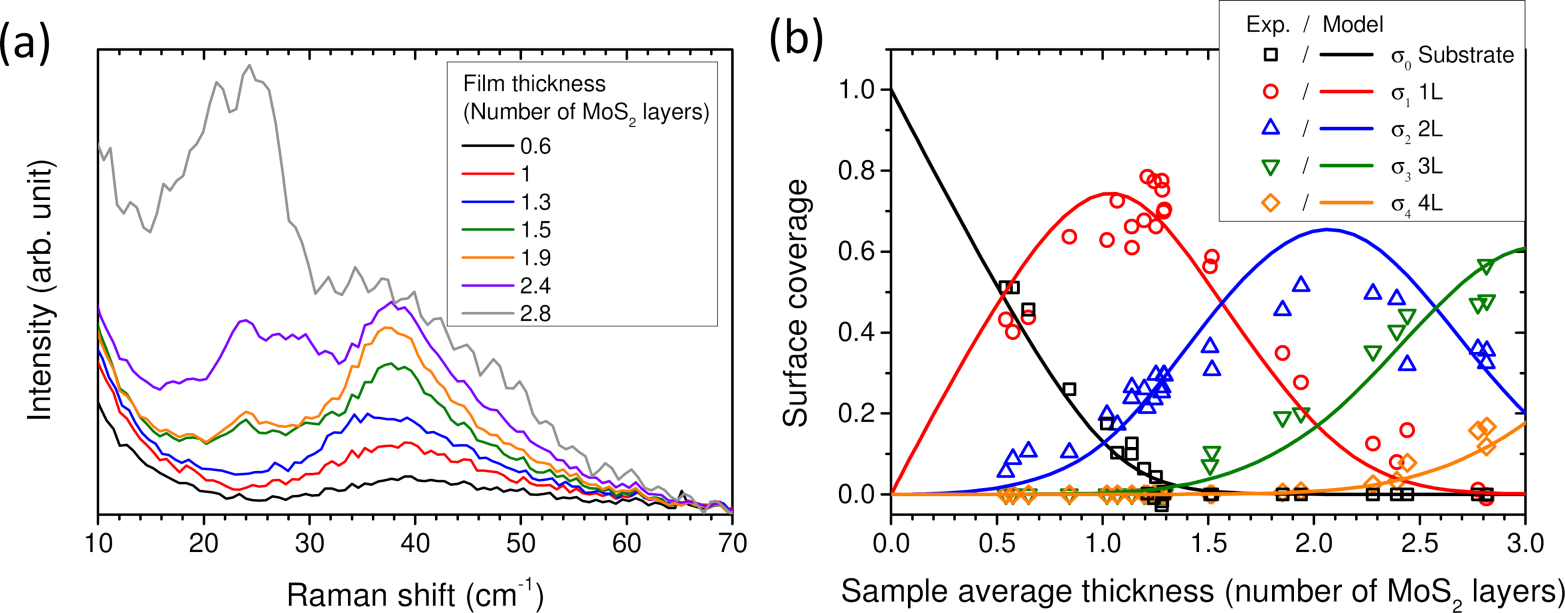
(a) Raman spectra in the ultralow-frequency region of selected DLI-PP-CVD samples with different average thicknesses as labelled in the legend. Presented spectra are averages extracted from 121-point Raman maps. (b) Surface coverages of bare substrate (black open squares), 1L-MoS_2_ (red open dots), 2L-MoS_2_ (blue open up-triangles), 3L-MoS_2_ (green open down-triangles), and 4L-MoS_2_ (orange open diamonds) as functions of the average DLI-PP-CVD sample thickness. The model results are shown as full lines. See the main text and Supporting Information File 1 for the description of the model.

To get further insight on the number of layer distributions in DLI-PP-CVD samples, we have measured their ULF modes. Representative ULF spectra are shown in Figure 9a for samples with average thicknesses ranging from 0.6 up to 2.8 MoS_2_ layers as estimated from *A*_2D_(Si)/*A*_0_(Si). Up to 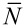 = 1.3, only the LB mode of 2L-MoS_2_ is observed around 40 cm^−1^ [20,38–41], showing that these samples can only be composed of 1L-MoS_2_ and twisted 2L-MoS_2_ plus possibly uncovered (bare substrate) regions. For thicker samples, the S mode of 2L-MoS_2_ around 24 cm^−1^ is additionally visible, as well as a signal between 25 and 30 cm^−1^, corresponding to the LB and S modes of 3L-MoS_2_ [32–33]. For 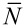 ≥ 2.4, the LB mode of 4L-MoS_2_ is also present around 21 cm^−1^; there may also be a weak signal around 17 cm^−1^ (corresponding to the LB mode of 5L-MoS_2_) reflecting the presence of 5L-MoS_2_. The S mode of 4L-MoS_2_ could be present as well around 28 cm^−1^, but it is hardly distinguishable from the LB and S modes of 3L-MoS_2_. Thus, ULF Raman spectra give valuable qualitative information on the different *N* present in each sample. Quantitative information relies on the determination of the surface coverages for each *N* (σ*_N_*), that is, the ratio between the surface covered by exactly *N* layers and the total surface. With *N* = 0 standing for the bare substrate and *N*_max_ being the largest number of layers present in the sample, the definition of the average number of layers 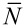 can be written as


[1] $$\bar{N}=\sum_{N=1}^{N_{\max}}\sigma_{N}\times N$$

and the total coverage (including bare substrate areas) is obviously 100%:


[2] $$\sum_{N=0}^{N_{\max}}\sigma_{N}=1$$

AFM imaging (see Supporting Information File 1, Figure S4) reveals that for 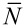 > 1.25, the surface is fully covered by MoS_2_, that is, σ_0_ = 0, which removes an unknown. In addition, for 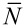 < 1.3, there is no signature of more than two layers, and we can set σ*_N_*_≥3_ = 0 with confidence. Hence, for 1.25 < 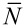 < 1.3, the set of Equations 1 and 2 simplifies to




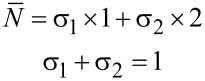




This allows one to readily determine the two remaining unknowns σ_1_ and σ_2_, since 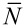 is known from *A*_2D_(Si)/*A*_0_(Si).

Hereafter, a linear relationship between the Raman signal 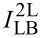, which is the LB mode peak intensity of 2L (*N* = 2) areas (the broad but well-identified 40 cm^−1^ peak), and the surface coverage is assumed, namely σ_2_ = 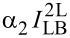. The ratio α_2_ = 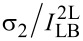 is determined from five samples (1.25 < 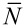 < 1.3) for which we now have both the coverage σ_2_ and the Raman signal 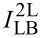.

Because α_2_ is now known and assuming that the linearity between σ_2_ and the Raman signal 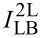 holds (which should be a good approximation for the thin multilayers considered here), σ_2_ = 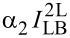 can be obtained directly for all samples from the Raman spectra, and is thus no longer an unknown.

We now turn to the samples with 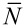 < 1.25, which may present some bare substrate areas, so σ_0_ and σ_1_ are a priori unknown. Both 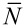 and σ_2_ are determined as explained above, and σ*_N_*_≥3_ = 0 is again a safe estimate. Hence, Equations 1 and 2 reduce to the system




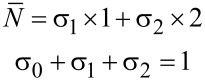




which may be solved trivially for σ_0_ and σ_1_.

A similar approach can be used for samples with 1.3 < 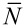 < 2 that are fully covered (σ_0_ = 0) and might present some trilayers but show no trace of thicker layers. We set σ*_N_*_≥4_ = 0, and the system of equations reduces to




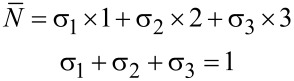




Both 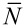 and σ_2_ are determined as explained above. Thus, the system can be trivially solved for the two remaining unknowns σ_1_ and σ_3_.

At this point it would be natural to get the proportionality between σ_3_ and a Raman signal attributed to 3L areas (*N* = 3), and proceed recursively to obtain σ_4_ in slightly thicker layers, and so on and so forth. In practice this becomes challenging because of the uncertainty on the 3L (*N* = 3) Raman signal, which is less clear than the 2L (*N* = 2) peak. Another approach gave better results.

Three samples with 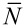 between 2.75 and 2.85 are thick enough to neglect σ_0_ and σ_1_, yet thin enough for σ_5_ to also be negligible as a first approximation. Equations 1 and 2 then reduce to




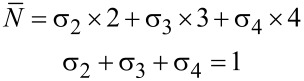




where 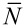 and σ_2_ are known, so σ_3_ and σ_4_ can be determined readily.

The LB mode of 4L-MoS_2_, located around 21 cm^−1^, is sufficiently separated from other modes to be identified (which was not the case for 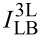), so that 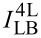 can be extracted from the spectra. From the three 2.75 < 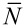 < 2.85 samples α_4_ = 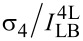 is determined. Then, assuming again a linear relationship σ_4_ = 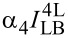, the coverage by 4L (*N* = 4) layers can be determined for all samples. This removes another unknown.

Now the last remaining case of 2 < 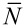 < 2.75 samples can be solved, as




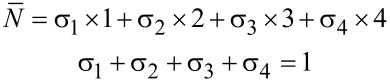




give σ_1_ and σ_3_ directly, since 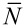, σ_2_ and σ_4_ are known.

The results obtained using this procedure are shown in Figure 9b where σ*_N_* (with *N* from 0 to 4) is plotted as a function of 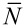, the average sample thickness. On this graph, all values of σ_2_ (respectively σ_4_) are calculated using 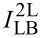 (respectively 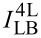) even for the samples used to derive the proportionality coefficient α_2_ (respectively α_4_). As shown in Figure 9b for samples with 1.25 < 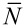 < 1.3 (respectively 2.75 < 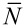 <2.85), we find by this way −0.03 < σ_0_ < 0.04 (respectively −0.01 < σ_1_ < 0.01) with little fluctuations around the expected value of 0.

Just below the full coverage of the sample surface by MoS_2_ (σ_0_ > 0), both σ_1_ and σ_2_ increase with a slight tendency of σ_2_ to increase faster than σ_1_. Indeed, 1L-MoS_2_ represents 80–90% of the deposited MoS_2_ for 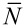 = 0.5 and 70–80% for 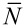 = 1.3. The maximum of σ_1_ is reached around 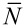 = 1.3 when the sample surface is totally covered by MoS_2_ (σ_0_ = 0), and σ_1_ starts to decrease above this value. Around 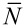 = 1.6, 1L-MoS_2_ only represents 50% of the MoS_2_. σ_2_ continues to increase and reaches a maximum value of ≈50% around 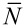 = 2 and then decreases for thicker samples. 3L-MoS_2_ starts to appear after the substrate surface is completely covered by MoS_2_ and increases continuously, representing about 50% of the thickest samples (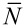 ≈ 2.8).

In order to verify our approach, we implemented a 2D growth toy model (see Supporting Information File 1 for details). The model results are shown in Figure 9b as full lines and give a good agreement with the experimental results. It should be noted that within this representation (σ*_N_* = *f*(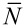)), the results of the model are remarkably robust to any parameter changes (the curves are almost insensitive to either doubling or halving the cell size and, thus, the advance rate, or to multiplying or dividing the growth rate by 5). In other words, this means that this comparison with the experiment cannot be used to validate any model parameters but demonstrates the relevance of the proposed procedure to estimate the σ*_N_* from the experiments. Nevertheless, it has to be noted that while for 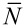 < 1.3 the ULF Raman signature of 2L-MoS_2_ remains very similar, it is not the case for thicker samples with the notable appearance of the S mode of 2L-MoS_2_ around 24 cm^−1^ [32–33]. This could mean that the stacking order distribution changes. As a consequence, the hypothesis based on the proportionality between 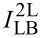 and σ_2_ would probably be less valid above 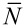 = 1.3, and an error on the absolute values deduced can be expected. However, the appearance of the S mode of 2L-MoS_2_ around 24 cm^−1^ could also be related to *N*L-MoS_2_ (with *N* ≥ 3) constituted of a stacking sequence where 2L are not twisted, for example, the so-called t(1+2)L, t(2+2)L, … structures [26,53]. In this case, our hypothesis would remain more appropriate. Despite this unknown as well as the other approximations made, we believe that the main tendencies can still be captured by the proposed analysis. Further works are needed to determine and improve the accuracy of the method.

## Conclusion

In this work we have reviewed all Raman information leading to the evaluation of the thickness of MoS_2_ flakes, that is, the layer number *N*. First, we have analyzed in detail the effects of some experimental parameters, namely the wavelength of the incident laser light used in the experiments, the power of the incident light, and the oxide thickness of the SiO_2_/Si substrate on which the flakes are deposited, on the quality and accuracy of Raman results. Based on this analysis, an experimental protocol has been defined and systematically applied to large MoS_2_ flakes (i.e., single-domain flakes much larger than the laser spot), including twisted MoS_2_ flakes, prepared by different methods on the one hand and to MoS_2_ thin films composed of nanoflakes prepared by the DLI-PP-CVD method on the other hand. Special attention was paid to the measurement statistics.

The limits of different Raman criteria which allow one to determine the thicknesses of MoS_2_ flakes, namely (i) the value of Δω_A−E_, (ii) the value of the normalized integrated intensity of A_1g_ and E^1^_2g_ MoS_2_ modes, and (iii) the value of the *A*_2D_(Si)/*A*_0_(Si) ratio, have been precisely studied in the different types of MoS_2_ samples. We definitely confirm that Δω_A−E_ cannot be considered a robust criterion to derive the number of layers in MoS_2_ samples. We found that the value of the *A*_2D_(Si)/*A*_0_(Si) ratio provides the most robust/reliable information to characterize the thickness of MoS_2_ large flakes, especially since it is found largely independent of the twist angle. The limit of application of this criterion is *N* ≤ 5, under the condition that the SiO_2_ thickness is precisely known.

We then apply this analysis procedure to DLI-PP-CVD samples constituted of nanoflakes with a lateral size of typically 50 nm (well below the laser spot size) with possibly a distribution of thicknesses and twist angles between adjacent layers of multilayer domains and a higher number of defects. Our results definitively establish the relevance of the *A*_2D_(Si)/*A*_0_(Si) ratio to give with good accuracy their average thickness 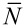, for 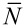 ≤ 3. Nevertheless, we emphasize that this criterion is not only related to the presence of MoS_2_ and can be influenced by several factors, such as the co-deposition of by-products or the presence of defects, leading to a wrong estimation of 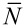. We propose to combine *A*_2D_(Si)/*A*_0_(Si) with the normalized integrated intensity of the MoS_2_ phonon modes, namely *A*(A_1g_) and/or *A*(E^1^_2g_). Although limiting the application to 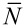 ≤ 3, this approach enables the validation of the *A*_2D_(Si)/*A*_0_(Si) ratio to determine 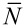 in the presented case, and we anticipate that it would avoid possible errors in unfavorable situations.

Finally, to get further insight on the number of layer distributions in DLI-PP-CVD samples, we have measured their ULF modes. An original procedure based of the measurement of the intensity of the layer breathing modes allows one to evaluate the surface coverage (σ*_N_*) for each *N*. A 2D growth toy model gives a good agreement with the experimental results supporting the proposed procedure to estimate the σ*_N_* from the ULF spectra.

## Experimental

### Samples preparation

#### Mechanical exfoliation

MoS_2_ flakes were obtained by micromechanical cleavage of a MoS_2_ crystal (HQ graphene) using scotch tape (Nitto) and PDMS slabs (Gel-pak). They were then transferred onto Si substrates with SiO_2_ layers of different thicknesses, namely 84, 87, 90, and 96 nm. Flakes were selected by optical microscopy and their thicknesses were determined by optical contrast.

#### Standard CVD process

MoS_2_ was grown by CVD on 87 nm SiO_2_ on Si substrates using MoO_3_ (Sigma-Aldrich, 25 mg) and sulfur (Sigma-Aldrich, 250 mg) powders as solid precursors using a 1 inch quartz tube furnace. MoO_3_ powder was placed in the center of the heating zone of the furnace, while sulfur was placed upstream at the furnace inlet. Prior to growth, air was evacuated by flowing Ar (ultrahigh purity, Linde) for 15 min at 200 sccm, after which the tube was heated to 200 °C for 10 min. The temperature was then increased to 750 °C under Ar (100 sccm), and it was held at this value for 15 min before cooling naturally to room temperature.

#### Direct-liquid injection pulsed-pressure chemical vapor deposition (DLI-PP-CVD)

The 12 × 11 mm SiO_2_/Si (with 87 nm or 96 nm SiO_2_ thicknesses) substrates were cleaned in acetone (C_3_H_6_O, technical, Acros Organics), isopropanol (C_3_H_7_OH, 99.8%, Höfer Chemie GmbH) and deionized water (H_2_O, Acros Organics) under ultrasonic agitation for 10 min each, before being blown dry with nitrogen. They were then immediately loaded on the susceptor of the reaction chamber (Annealsys MC-050) for deposition. Solutions of 0.001 M molybdenum hexacarbonyl (Mo(CO)_6_, 98%, Strem Chemicals) and 0.002 M sulfur (S, 99.999%, Acros Organics) in anhydrous toluene (C_6_H_5_CH_3_, 99.8%, Sigma-Aldrich) were used as precursors. The process is as follows: Following sample installation, the chamber is closed and brought to about 0.02 mbar. For monolayer depositions, it is imperative that the substrate is thoroughly cleaned and free of adsorbates. Therefore, to ensure complete desorption of remaining contaminants, the samples were kept for 30 min under vacuum at room temperature inside the deposition chamber. For the first part of the process, the pumping direction is reversed so that all species are pumped from the deposition chamber to the back of the reactor.

Nitrogen (800 sccm) is flowed through the chamber (200 sccm through the gas line, and 600 sccm through two injection heads) and the substrates are brought to 750 °C at a ramp of 2 °C/s. The reactor is kept in this state for 5 min for homogenization purposes. While still in reverse direction pumping, 0.3 g/min of both precursors are injected and vaporized to prepare the evaporation system for deposition. Then, the Mo(CO)_6_ injection is stopped, the pumping direction is switched back to the deposition direction and hydrogen (40 sccm) is added to the gas mix. For 1 min, sulfur is injected to clean any remaining contaminants, and to prepare the surface of the substrate for MoS_2_ deposition, then the deposition works in 20 s cycles. During one cycle, a single pulse of 3 to 10 ms of Mo(CO)_6_ is injected while the S injection is set to 0.3 g/min. This 20 s cycle is repeated 80 to 160 times. The quantity of MoS_2_ deposited is controlled by the quantity of Mo(CO)_6_ injected, that is, the pulse duration and the number of cycles.

### Raman spectroscopy

Raman spectra and maps were recorded using an Acton spectrometer fitted with a Pylon CCD detector and a 1800 grooves/mm grating (≈0.6 cm^−1^ between each CCD pixel). The samples were excited with a 532 nm (2.33 eV) laser (Newport Millennia Prime or Cobolt Samba) throughan Olympus microscope objective either 100× (numerical aperture 0.9) or 50× (numerical aperture 0.5). The full width at half-maximum (FWHM) of the focused laser spot with the 100× objective is about 380 nm. Optimized focus conditions were checked for each measurement. The samples were mounted on a three-axis piezoelectric stage (Physik Instrumente) to ensure the precise positioning and focusing of the laser spot. A Si(111) wafer with only native oxide sample was used as a daily reference for the system. The laser power was continuously measured during acquisitions allowing for intensity normalizations of the Raman spectra at each point of the maps. All data presented in this paper, unless specified otherwise, are extracted from Raman maps constituted by hundreds to thousands points (see Supporting Information File 1 for an example), which were analyzed using a custom-made software. All reported points are the average values obtained by Gaussian fitting of the data distribution extracted from Raman maps (corresponding to hundreds to several thousands of spectra), and the error bars correspond to 99.7% confidence intervals (±3 standard deviations).

### 2D growth toy model

The model used the DynamicGrids.jl package, which was part of the Dispersal.jl framework [54], see Supporting Information File 1 for more details.

## Supporting Information

Supporting Information File 1 contains additional figures with an example of Raman maps, the Si mode as a function of the laser power, a comparison between two microscope objectives, other intensity references, atomic force microscopy images, and details of the 2D growth toy model. Supporting Information File 2 is a recording of the growth simulation.

File 1 Additional experimental data.

File 2 Recording of the growth simulation.
