# Supplementary material for: Determining by Raman spectroscopy the average thickness and N-layer-specific surface coverages of MoS2 thin films with domains much smaller than the laser spot size
Source: Beilstein J Nanotechnol. 2024 Mar 7;15:279–96. doi: 10.3762/bjnano.15.26 (PMC10928926; doi:10.3762/bjnano.15.26)
Supplement: File 1 — Additional experimental data. [file Beilstein_J_Nanotechnol-15-279-s001.pdf]

BEILSTEIN JOURNAL OF NANOTECHNOLOGY

Supporting Information

for

Determining by Raman spectroscopy the average thickness and *N*-layer-specific surface coverages of MoS_2 thin films with domains much smaller than the laser spot size

Felipe Wasem Klein, Jean-Roch Huntzinger, Vincent Astié, Damien Voiry, Romain Parret, Houssine Makhlouf, Sandrine Juillaguet, Jean-Manuel Decams, Sylvie Contreras, Périne Landois, Ahmed-Azmi Zahab, Jean-Louis Sauvajol and Matthieu Paillet

Beilstein J. Nanotechnol. **2024,** *15,* 279–296. doi:10.3762/bjnano.15.26

Additional experimental data

License and Terms: This is a supporting information file under the terms of the Creative Commons Attribution License (https://creativecommons.org/licenses/by/4.0). Please note that the reuse, redistribution and reproduction in particular requires that the author(s) and source are credited and that individual graphics may be subject to special legal provisions.
The license is subject to the *Beilstein Journal of Nanotechnology* terms and conditions: (https://www.beilstein-journals.org/bjnano/terms)

1. Example of Raman maps

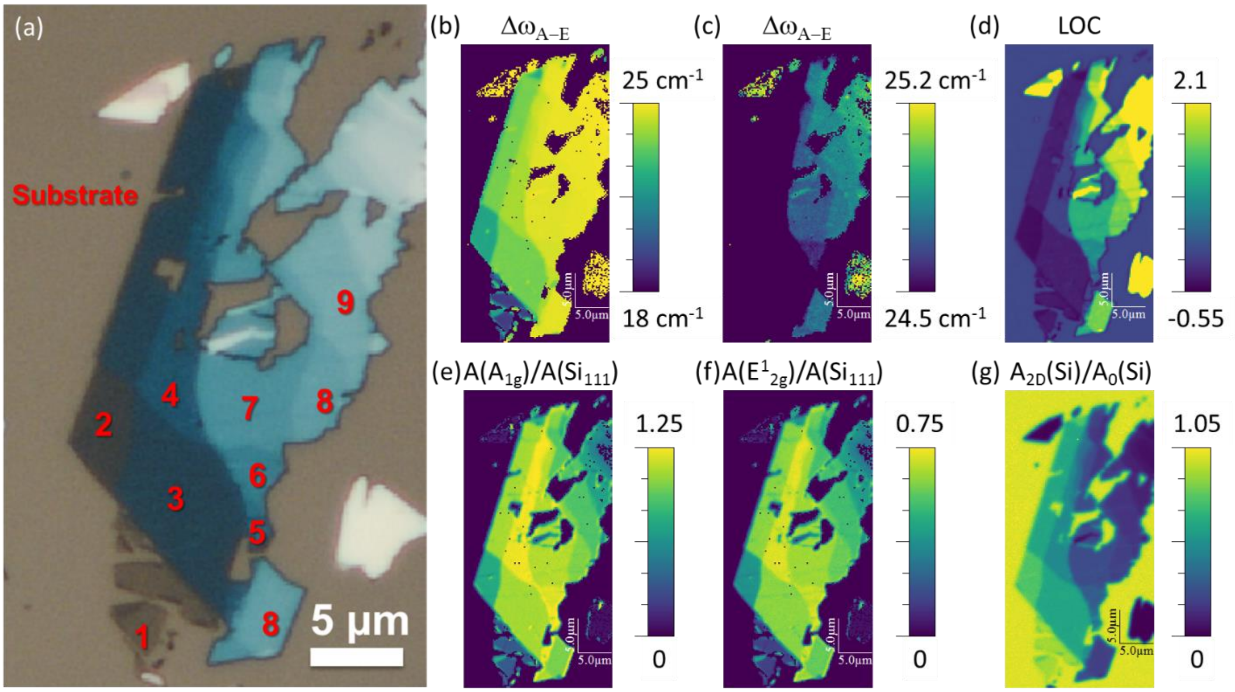

Figure S1: (a) Optical microscopy picture of a mechanically exfoliated MoS_2 sample on a 84 nm SiO_2/Si substrate. The substrate and the MoS_2 regions with a number of layers from 1 to 9 as determined by ULF Raman and spectral microreflectivity are labelled in red. (b) and (c) corresponding Raman map of the frequency difference between the A_{1g} and E^1_{2g} MoS_2 phonon modes with two different color scales as shown on their right hand side. (d) Laser optical contrast map. (e)-(g) Raman maps of the normalized integrated intensities of (e) the A_{1g} MoS_2 phonon mode, (f) the E^1_{2g} MoS_2 phonon mode and (g) the Si 521 cm^{-1} mode. The A_{1g} (e) and E^1_{2g} (f) maps are normalized with an external reference which is a bare Si(111) wafer with only native oxide. The Si map (g) is normalized with the bare substrate appearing in yellow. Z-scale colorbars are shown on the right hand side of each map.

2. Si mode as a function of the laser power

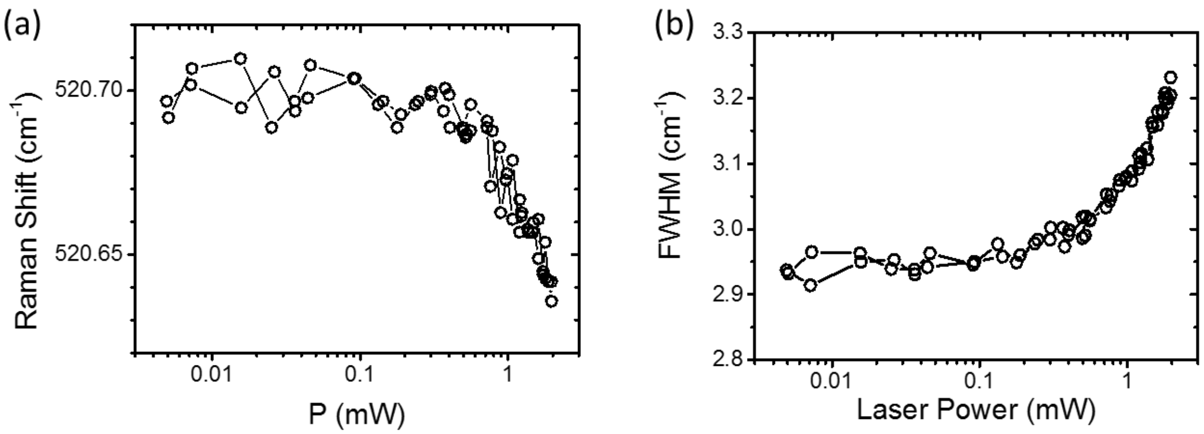

Figure S2: Evolution of the Si 521 cm^{-1} Raman mode frequency (a) and full width at half maximum (b) as a function of the incident laser power during a cycle from 5 µW up to 2 mW and back to 5 µW.

3. Other intensity references

In the main text, the MoS_2 A_{1g} and E^1_{2g} modes integrated intensities are normalized relatively to the integrated intensity of the 521 cm^{-1} mode of a bare Si(111) wafer with only native oxide. In the literature or in future works other intensity references can be considered and Raman set-ups with different characteristics can be used. To enable results comparison, we have measured i) bulk MoS_2 crystal (HQ graphene) for which we found, $A(A_{1g})/A(Si_{111}) = 0.116$ and $A(E^1_{2g})/A(Si_{111}) = 0.067$, ii) the 521 cm^{-1} mode of Si(100) with 90 ± 6 nm SiO_2 (resp. native oxide) which integrated intensity equals 1.32 ± 0.05 (resp. 0.68) relative to Si(111) and iii) the polarization ratio of our collection and detection system which is H:V=1:0.76, with H (resp. V) standing for horizontal (resp. vertical) which is parallel (resp. perpendicular) to laser polarization.

4. Comparison between two microscope objectives

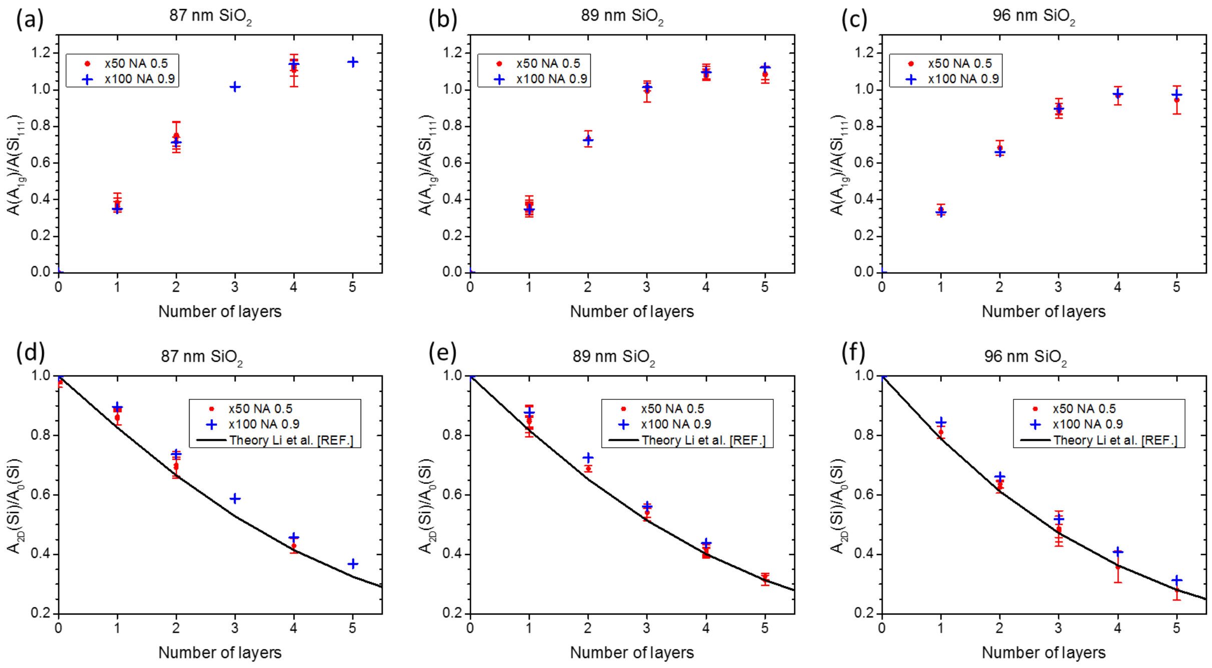

Figure S3: Comparison between x50 objective with N.A. 0.5 (red filled dots) and x100 objective with N.A. 0.9 (blue crosses), dependence with the number of MoS_2 layers of the normalized integrated intensities (see text) of the A_{1g} MoS_2 mode (a-c) and of the Si 521 cm^{-1} mode (d-f). Data for three different substrate SiO_2 thicknesses are presented: (a) and (d) 87 nm, (b) and (e) 89 nm and (c) and (f) 96 nm. For the 100x objective, points correspond to the average values of Figure 3 in the main text. Black lines in (d-f) are extrapolation for the corresponding SiO_2 thicknesses of the semi-empirical model for an objective N.A. of 0.45 of Li *et al.* [1].

5. Atomic force microscopy (AFM) images

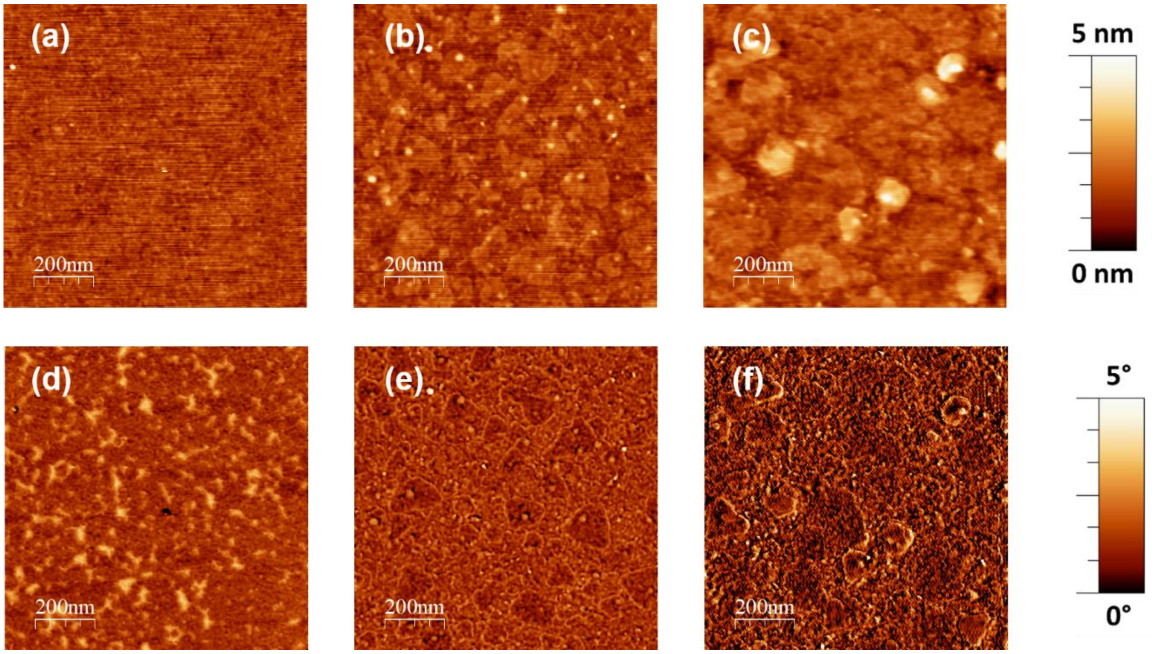

Figure S4: Representative AFM images of DLI-PP-CVD samples with $\overline{N}$ < 1.25 ((a) and (d)), 1.25 < $\overline{N}$ < 1.3 ((b) and (e)) and 1.3 < $\overline{N}$ < 2 ((c) and (f)). (a), (b) and (c) topological and (d), (e) and (f) phase mode AFM images with corresponding colorbars on the right hand side. In (d), the lighter areas (higher phase) coincide with bare substrate regions (*i.e.* not covered by MoS_2). Such phase contrast is absent on the DLI-PP-CVD samples with $\overline{N}$ > 1.25 as illustrated in (e) and (f) which are fully MoS_2 covered.

6. 2D growth toy model

Contrary to standard CVD growth of MoS_2, the DLI samples were produced rather quickly with a large amount of precursors, and the samples have small domain sizes (around 50 nm). The model is thus based on a simplifying assumptions[1]:

1) Once a flake has nucleated, the advance rate of the growth front[2] $\alpha_l = 1\ \mathrm{nm}/s$ is constant (only dictated by the incorporation to the flake edge). When another flake is met the advance stops.
2) The germination rate (density of probability of germination) $g = 1 \times 10^{-4}\ \mathrm{nm}^{-2}\mathrm{s}^{-1}$ is constant.

The “high-performance grid-based spatial simulation framework” DynamicGrids.jl [3,4] was chosen for its speed and simplicity. It is part of the Dispersal.jl framework [3]. At the time of writing only square grids were readily available, but the goal was only to show why multilayers seemed to “wait for the previous layer to grow” before appearing. The model shows that it is possible to qualitatively reproduce these observations with constant probabilities. The resulting shapes are squared instead of hexagonal or triangular, but that changes only details such as the relation between characteristic size and perimeter or

---

[1]Ref. [2] also used a constant advance rate, surface diffusion seemed fast enough to be ignored even for micrometer-sized flakes in the conditions they considered.

[2]Ref. [2] Kinetic Monte Carlo model also showed a “line-by-line” growth when the flake is large enough for the atomistic irregularity of the edge to be indiscernible. In our case, it is the “collisions” with other nano-domains that lead to irregular shapes and smooth out the curves.

area. Those details would be important if the goal was to extract quantitative values of the parameters, which is out of scope here (much more elaborate models exist, see *e.g.* [2]). The sample area is divided in $N_r \times N_c$ cells on a square grid. Each cell holds the number of layers covering it. So at each time step (time t), the sample is described by a $N_r \times N_c$ matrix of integers.

The matrix is updated for the next time step (time $t + dt$) as follow, for each cell:

1) If at time t any of the cell first neighbors is higher than the cell (meaning there is a growth edge nearby), then increment the cell. So the nearby edge advances to the cell. Note: it does not matter if there are several edges nearby; the cell is incremented only once.
2) Otherwise, randomly increment the cell by 1, with a fixed probability $P_g = ga^2dt$. This crudely models the germination process.

The time increment $dt = 1\,s$, and the cell size $a = 1\,\mathrm{nm}$ define the linear advance rate $\alpha_l = a/dt$. For a 2 min process (120 s) on a 2000x2000 grid the calculation takes less than a second on an AMD Ryzen 9 3900X 12-Core processor. A recording of the growth is available as “growth.mp4”.

Of course, there are some run to run fluctuations in the coverage evolution, which would be akin to fluctuations from one sample position to another. But fluctuations happen to be negligible in this case, and the grid area (2 µm square) is still smaller than the probed area (the spectra are averaged over several spot sizes to increase signal to noise ratio). This would have to be reconsidered if the spatial resolution were not limited by diffraction (with a tip-enhanced Raman spectroscopy probe for instance), or if the domain sizes were larger (fewer domains probed at once).

This set of parameters seemed reasonable and gave an acceptable agreement with the experiments.

The curves in the following figure are remarkably robust to any parameters change (neither doubling or halving the cell size, and thus the advance rate, nor multiplying or dividing by 5 the growth rate).

In the following, the capital N stands for number of layers, while the lower case n_N stands for the "number of cells that are covered by exactly N layers". We'll call n_t the total number of cells used in the calculation. With these definitions, the coverage by exactly N layers is $\sigma_N = \frac{n_N}{n_t}$ and the average number of layers is $\langle N \rangle = \frac{1}{n_t} \sum_N N \times n_N = \sum_N N \times \sigma_N$. Since initially the multilayers are scarce, the $N \geq 2$ terms are negligible and $\langle N \rangle \sim \sigma_1$. This explains why close to 0 the $\sigma_1(\langle N \rangle)$ slope is 1, and $\sigma_0(\langle N \rangle) \sim 1 - \langle N \rangle$. Two effects tend to reduce the slope of $\sigma_1(\langle N \rangle)$: (i) the growth of the second layer reduces σ_1, since a cell with two layers is no longer counted as a monolayer. (ii) at some point a monolayer flake encounters other monolayers, which limits their expansion. This is why monolayers eventually get covered entirely ($\sigma_1 \rightarrow 0$), despite the fact that all layers have the same advance rate in our simplified model.

This model also provides some support to the Raman scattering analysis made in the article core as shown in Figure S5. For $\langle N \rangle \in [1.25,\ 1.3]$ the $N = 1$ and $N = 2$ contributions indeed dominate. For $\langle N \rangle \in [1.3,\ 2]$ the $N = 3$ contribution rises. For $\langle N \rangle \in [2,\ 2.75]$ the $N = 1$ or the $N = 4$ contributions are to be taken into account, in addition to the $N = 2$ and $N = 3$. This most challenging region is thus treated last in the proposed analysis. Finally, for $\langle N \rangle \in [2.75,\ 2.85]$ both the modeled $N = 1$ and $N = 5$ contributions are negligible.

So the assumptions underlying the proposed procedure are valid in the model.

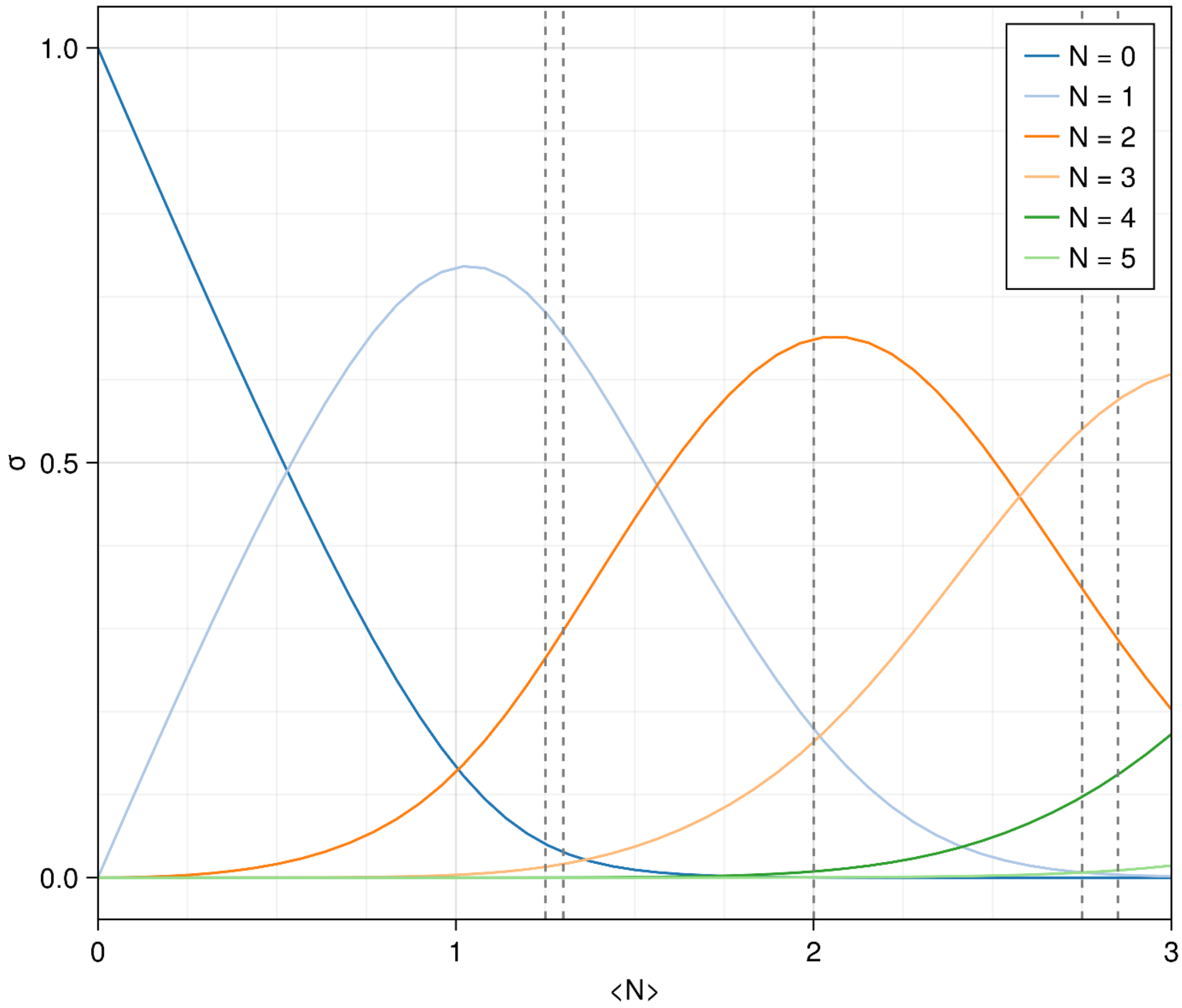

Figure S5: Coverage σ_N (ratio of the surface covered by exactly *N* layers to the total surface) as a function of the average number of layers. The vertical dashed lines mark the interval bounds used in the main article (1.25, 1.3, 2, 2.75, 2.85).

7. References

[1] Li, X.-L.; Qiao, X.-F.; Han, W.P.; Zhang, X.; Tan, Q.-H.; Chen, T.; Tan, P.-H. *Nanotechnology* **2016**, *27*, 145704. doi:10.1088/0957-4484/27/14/145704

[2] Govind Rajan, A.; Warner, J. H.; Blankschtein, D.; Strano, M. S. *ACS Nano* **2016**, 10, 4330–4344. doi:10.1021/acsnano.5b07916

[3] Maino, J. L.; Schouten, R.; Umina, P. *J. Appl. Ecol.* **2021** 58, 789–800. doi:10.1111/1365-2664.13812

[4] https://github.com/cesaraustralia/DynamicGrids.jl
